# Genetic correlations of direct and indirect genetic components of social dominance with fitness and morphology traits in cattle

**DOI:** 10.1186/s12711-023-00845-8

**Published:** 2023-11-30

**Authors:** Beniamino Tuliozi, Roberto Mantovani, Ivana Schoepf, Shogo Tsuruta, Enrico Mancin, Cristina Sartori

**Affiliations:** 1 Department of Biology, Duke University, Durham, NC 27708 USA; 2 Department of Agronomy, Food, Natural Resources, Animals and Environment, University of Padova, Viale Dell’Università 16, 35020 Legnaro, Italy; 3 Department of Sciences, Augustana Campus, University of Alberta, 4901 46 Ave, Camrose, AB T4V 2R3 Canada; 4 Department of Animal and Dairy Science, University of Georgia, Athens, GA 30602 USA

## Abstract

**Background:**

Within the same species, individuals show marked variation in their social dominance. Studies on a handful of populations have indicated heritable genetic variation for this trait, which is determined by both the genetic background of the individual (direct genetic effect) and of its opponent (indirect genetic effect). However, the evolutionary consequences of selection for this trait are largely speculative, as it is not a usual target of selection in livestock populations. Moreover, studying social dominance presents the challenge of working with a phenotype with a mean value that cannot change in the population, as for every winner of an agonistic interaction there will necessarily be a loser. Thus, to investigate what could be the evolutionary response to selection for social dominance, it is necessary to focus on traits that might be correlated with it. This study investigated the genetic correlations of social dominance, both direct and indirect, with several morphology and fitness traits. We used a dataset of agonistic contests involving cattle (*Bos taurus*): during these contests, pairs of cows compete in ritualized interactions to assess social dominance. The outcomes of 37,996 dominance interactions performed by 8789 cows over 20 years were combined with individual data for fertility, mammary health, milk yield and morphology and analysed using bivariate animal models including indirect genetic effects.

**Results:**

We found that winning agonistic interactions has a positive genetic correlation with more developed frontal muscle mass, lower fertility, and poorer udder health. We also discovered that the trends of changes in the estimated breeding values of social dominance, udder health and more developed muscle mass were consistent with selection for social dominance in the population.

**Conclusions:**

We present evidence that social dominance is genetically correlated with fitness traits, as well as empirical evidence of the possible evolutionary trade-offs between these traits. We show that it is feasible to estimate genetic correlations involving dyadic social traits.

**Supplementary Information:**

The online version contains supplementary material available at 10.1186/s12711-023-00845-8.

## Background

An individual’s phenotype is determined not only by its own genetic background, but also by the genetic backgrounds of its interacting counterparts [1, 2]. Indirect genetic effects (IGE), i.e. the influence that the genotype of conspecifics has on the focal individual phenotype, are used to quantify the effects that the social environment has on the expression of behavioral, morphological, and life-history traits [3, 4]. To date, IGE have been linked to the expression of several traits, including seasonal reproductive timing [5–7], competition for resources [8], aggressiveness [9, 10], and social dominance [11, 12].

The evolutionary trajectory of social dominance, or ‘a dyadic social relationship that emerges from sequences of agonistic interactions, where one individual exhibits subordination’ as defined by Strauss et al. [13], is especially suitable for study in the light of IGE. Competition in several species is in fact resolved by agonistic interactions that, when repeated over time, lead to the establishment of social hierarchies. These interactions often result in clearly defined winners and losers: the outcomes of these contests are usually modelled by assigning 1 to the winner and 0 to the loser. Such outcomes may mediate prime access to limited resources and mating opportunities, and for this reason, social dominance is considered a key determinant of fitness. As the outcome of each agonistic interaction depends on the phenotype of both individuals, to investigate the genetic architecture of social dominance it is necessary to consider both components of the trait: the genotype of the focal individual as the direct genetic effect, and the genotype of its opponent as the IGE [14].

Whenever IGE are considered in studies of dyadic contests with a win/lose outcome as a dominance trait, the total genetic variance is the sum of the direct genetic effect, the indirect genetic effect, and twice their covariance. In 1/0 contests, since for each direct winner there is necessarily an indirect loser and vice versa, the direct and indirect genetic variances and their covariance are all equal in magnitude, but the covariance has a negative sign. This postulates that the net genetic variance associated with social dominance, i.e., the genetic variance available for selection of the trait, is zero. The few empirical studies that have focused on the IGE underlying social dominance [11, 12] have accordingly found a genetic correlation of − 1 between the direct and indirect components of social dominance. Furthermore, this relationship leads to a total heritability of the trait of about 0. This apparently straightforward result has an important real-life consequence: even when individuals in a population are consistently selected for social dominance, there is a constraint on the evolution of the mean observed phenotype. As the “winning” phenotype spreads in the population, it creates an environment where competition is exacerbated, resulting in no mean increase in the winning rate (“treadmill of competition” [15]). In other words, more and more dominant individuals (winners) are selected over time, but they find themselves competing in an environment full of other winners.

There is another aspect that is rarely investigated and further complicates this framework: different traits might be intricately connected and social dominance (its direct component), for example, can correlate with traits that allow individuals to physically overcome their opponents, including armament size [16], fighting ability [17, 18] or aggressiveness [19–21]. Traits can also be connected through trade-offs, with functional constraints resulting in negative genetic correlations between fitness components [16, 22]. However, as greater dominance often results in greater resource acquisition, in a natural population we would expect individuals that are more dominant to be able to increase their health and size, leading to a positive association between these traits and social dominance [21, 23]. Thus, many genetic trade-offs could be masked in wild populations, as dominance is key to successful reproduction and resource acquisition [24–26]. And yet, in other populations, trade-offs have indeed been found and can result in dominant individuals having poorer values for traits such as parental care [27], survival [28, 29] or fertility [30]. These antagonistic relationships between traits are especially observed for female dominance traits, where in spite of the advantages of high social ranks (see e.g., [31]), the energy allocated to maintain a dominant position depresses individual fertility, increases the risk of abortion, and reduces offspring survival [32]. Studying a population of farmed animals, for which (generally) resources are unlimited, allows to exclude processes such as competitive ability that co-varies positively with resource-dependent traits [33]. For this reason, in livestock populations it is easier to find genetic correlations that underlie the presence of genetic trade-offs, as they are not confounded by variable resource acquisition. Rare empirical evidence of unfavourable genetic correlations between social dominance-related traits and health and life history traits is found for fighting ability of cows (*Bos taurus*), which is correlated with traits linked with morphological and physiological masculinization, but it is also negatively correlated with fertility, milk production and longevity [26, 34, 35]. This means that, in theory, a continued selection for winners of social contests could unfavourably impact these traits, by means of their genetic correlations with factors that cause social dominance via, for example, pleiotropic effects. To date, all genetic correlations between social dominance and other traits (whether antagonistic or not) have only been estimated for the direct component of social dominance, but not for both the direct and indirect components.

Yet, correlations between both direct and indirect genetic effects of social dominance and antagonistic fitness and health traits are required for a comprehensive understanding of the evolutionary processes that underlie social dominance [33], particularly as they might allow us to detect novel and complex evolutionary trajectories in these traits. In nature, traits may also evolve, but mean fitness may not (see e.g., [16]), and one reason could be a correlated evolution of the social environment, which is a form of transmission bias [36, 37]. In the context of social dominance, if winners were selected, the trait might evolve, but there would be no change in the average social dominance phenotype. Thus, we would not be able to find any effect of selection when analysing the ‘social dominance’ phenotype. However, this might not be the case if we modelled a morphological or life history trait that is correlated to social dominance, such as fertility (as suggested by [33]). If a correlation between social dominance and life history, health, or morphological traits was found, then selecting winners in a population would potentially impact the correlated traits. While we would not observe an effect of selection on social dominance itself (as it would be masked by IGE), dominant individuals would still be selected, and this could potentially lead, for example, to the selection of less fertile animals, with physiological and morphological characteristics that are more suitable for winning fights.

To our knowledge, a multivariate analysis investigating the associations of both the direct and indirect additive genetic effects of social dominance with life-history traits has not been attempted [2], but see [38] for a correlation between exploratory behaviour and both direct and indirect genetic components of aggression. To be run, such a model would indeed require complex calculations [39], an enormous amount of data (typically a long-term dataset) and an extensive pedigreed population [40].

In the present study, we used agonistic contest data that were collected over 20 years from a breeding population of the Aosta Chestnut-Black Pied cattle breed to investigate the direct and indirect genetic components of social dominance. In the Aosta region of Italy, where this breed is native, pairs of cows traditionally compete for social dominance in ritualized bloodless interactions (called ‘Batailles des Reines’) [17, 41]. Contests between cows follow the same dynamics that are naturally encountered in herds at summer pastures when unfamiliar individuals meet and compete to establish dominance hierarchies [42]. In addition, fertility, morphology, and milk production are routinely measured in the entire population of this breed. We investigated correlations between the aforementioned traits, which include key fitness traits, and the direct and indirect components of social dominance by performing a bivariate animal model analysis. We used the variance-partitioning approach [43–45] to distinguish between direct and indirect genetic variances of social dominance and to account for their covariance. Because this method also allows to model the non-genetic (permanent environmental) component of indirect effects (in addition to the IGE), it also prevents biases in direct and indirect variances [33, 46]. Intriguingly, the individuals in this population are actively ranked and selected based on their victories in the contests, thus creating a system where social dominance is under positive selection. While the ‘social dominance’ phenotype cannot change, we do expect genetic change in the population, as genotypes associated with winning are selected for generation after generation. This not only means that the estimated breeding values (EBV, the individual genetic merit of a trait) for social dominance might have increased in the past decades, but also that all traits correlated with social dominance might have been ‘dragged’ along and evolved, although they are not under direct selection. The peculiar tradition of the ‘Batailles des Reines’ creates a unique set-up, that allows the study of the evolutionary consequences of selection for a social trait. Thus, we estimated the evolutionary trajectories of all traits (and of their genetic correlations) over 20 years to test how the direct and indirect genetic correlations between social dominance and fitness traits would influence the rate of evolutionary change in quantitative traits [47, 48].

## Methods

### Individuals studied and data collection

All studied individuals were cows of the Aosta Chestnut-Black Pied cattle (*B. taurus*) breed, a local population native of the western Alps with approximately 12,300 heads (www.fao.org/dad-is). The cows are raised in different herds, each housed on a farm from fall to spring, while in summer each herd goes to pasture in Alpine valleys. This breed is selected for three production purposes: milk, meat and fighting ability, all included in the selection index. Indeed, this breed is traditionally used in a series of contests, the ‘Batailles des Reines’ (Battles of the Queens, here simply referred as ‘Batailles’), mimicking the traditional establishment of dominance hierarchies that occur at summer pasture. Two cows are led to face each other and are free to interact until dominance is asserted when either adopts a submissive posture—thus, every duel results in a winner and a defeated individual. Most of the contests are resolved by non-physical interactions or quick contacts: social dominance interactions in this species almost never lead to all-out aggression, as ritualized displays of submission and dominance allow to establish social hierarchies while reducing aggression. Welfare of the animals is guaranteed by qualified expert veterinarians who are always present.

‘Batailles’ happen weekly, with 21 to 22 different day-long competitions per year, occurring between April and October (see [17, 41] for more details). All the cows are divided into three weight categories, with duels happening only within category. Each cow is allowed to compete in multiple daily competitions per year, but winners of a day-long competition are not generally part of the remaining competitions for the year. Within each day-long competition, cows can engage in up to seven knock-out duels, with the losing individuals being eliminated from the daily competitions. Although possible, it is very rare for cows to face familiar individuals (i.e., from the same herd or that they have faced before), given the large number of herds and interactions.

### Target traits and models

We analysed social dominance (see details in the following paragraph), as well as its correlation to other traits that are routinely recorded in this breed: fertility, milk yield, somatic cell count (SCC), and seven morphological traits. The fertility trait used here is the parturition-conception interval (in months), i.e., the timespan between a parturition and the subsequent conception date. It is the most widely used measure of fertility in animal breeding (e.g., [49]), with longer intervals being associated with less fertile cows due to events such as abortion, disease, or missed conception causing the loss of a reproductive season. Data on milk yield consist in individual test-day (TD) records. Somatic cells are body-derived cells and an indicator of the inflammatory status of the mammary gland [50], which typically appear after mastitis events. They are counted with an automatic cell counter and their measurement (somatic cell count, SCC) is considered an udder health indicator (as in, e.g., [48]) and is analysed as a somatic cell score (SCS; [51]), i.e. SCS = 3 + (log2(SCC/100 000 cells/mL)]. Higher values of the SCS (and thus of the number of somatic cells) indicate poorer udder health. We also used seven morphological traits (MT) that were recorded during the annual scoring of primiparous cows for linear type traits. The evaluation for linear type traits is carried out once over the life of cows (at the age of about 3 years) by trained classifiers following a 1 to 5 points scale with extreme points corresponding to biological extreme values for that trait (see also [52]). Our study considered the following traits: (i) fore udder attach (size of the fore udder); (ii) rear udder attach (distance between skinfolds and the line joining ischium and hock); (iii) udder width; (iv) overall udder score (an evaluation of udder size); (v) thinness (general evaluation of bone and muscle structure); (vi) thorax depth (distance between the top line of the chest and the chest floor); and (vii) front muscularity (muscle mass around neck and shoulders). These seven morphological traits were retained because of their role as indicators of a “female-like” or “male-like” morphology, as in Sartori et al. [34]. High scores for thinness and udder measurements identify a female-like conformation, whereas high values for front muscularity and thorax depth identify a more male-like bone structure, and have been associated with fighting ability [34].

Data were provided by the National Breeders Association (ANABoRaVa, www.anaborava.it) and by the Regional Farmer Organization (AREV; www.arev.it). The dataset spanned 20 years, from 2000 to 2019 (but from 2000 to 2020 for MT). Pedigree data covered a timespan of 14 generations and were obtained from the herdbook of the Aosta Chestnut-Black Pied population, dating back to 1955. Linear single-trait mixed animal models were first run for each trait to estimate variance components. Detailed information on these traits and on the single-trait models used for estimating their variance components are provided in Additional file 1 and Additional file 2: Table S1.

### Social dominance

The identity of all pairs of cows that engaged in a dyadic interaction (a duel) during a day of ‘Batailles’, the outcome of each duel, the individual weights and the related weight category were routinely collected by the association responsible for the ‘Batailles’ (amisdereines.it) and sent to the National Breeders Association at the end of each annual tournament. We considered each dyadic interaction between cows as a record, following Sartori and Mantovani [12]. To avoid data duplication, all dyadic interactions were considered only once, with one individual being randomly chosen as focal while the other was recorded as opponent: in other words, each interaction appears only once in the dataset. The individual phenotype for each dyadic interaction was thus a binomial trait assuming the values of either 1 for a victory or 0 for a defeat. To perform the analysis, we retained only dyadic interactions of individuals that appeared in the dataset at least once as focal and once as opponent. This was necessary because the software used (THRGIBBSF90, see below) requires all individuals to exert both a direct and an indirect genetic effect on at least one phenotypic record (no individual can appear in the dataset only as focal or only as opponent). Starting from 47,641 duels, we retained 37,996 duels that involved 8789 animals, with 19,707 animals present in the pedigree. The mean additive relatedness of the pairs of fighting cows was negligible (relatedness coefficient of 0.008).

Fixed effects retained in the analysis for social dominance were: year (19 levels), day of tournament (21–22 levels, from 1 to 21st or 22nd day of yearly competition, from the 3rd Sunday of March to the 3rd Sunday of October), weight category (3 levels with average weights of 500, 550 and 650 kg), difference in age between the focal individual and the opponent (categorized in 13 classes, with the central class being 0 and representing equal age, as in [12]) and weight differences (categorized in 7 classes, with the central class being 0 and representing equal weight). Note that while not all fixed effects affect the estimation of the variance component in social dominance (because the mean phenotype is constant), a model without fixed effects ranks the EBV of cows differently than a model with fixed effects. The herd, i.e., the farming environment where the animals are raised (761 levels) was included in the analysis as two random effects, i.e., herd of the focal and herd of the opponent individuals. Several preliminary models were run to decide to retain the abovementioned effects. Given that, in the dataset, all fights are present only once, it was not necessary to use correlated residuals.

In the final model, considering a dyadic interaction $k$ performed by a focal individual $i$ vs an opponent $j$, the binary outcome ${y}_{ijk}$ (0 for defeat and 1 for win) was distributed as:

$$ y_{ijk} \sim {\text{ B}}\left( {logit^{ - 1} \left( {l_{ijk} } \right)} \right), $$

where $\text{B}$ is the Bernoulli distribution, ${l}_{ijk}$ is the liability on the logit scale of the event “$i$ wins the duel”, thus resulting as dominant. The ${l}_{ijk}$ was predicted using the following model [12] including both additive genetic and permanent environmental components:

$$ {\mathbf{l}} = {\mathbf{X }}{\varvec{\upbeta}} + {\mathbf{W}}_{{\mathbf{D}}} {\mathbf{Pe}}_{{\mathbf{D}}} + {\mathbf{W}}_{{\mathbf{C}}} {\mathbf{Pe}}_{{\mathbf{C}}} + {\mathbf{Z}}_{{\mathbf{D}}} {\mathbf{a}}_{{\mathbf{D}}} + {\mathbf{Z}}_{{\mathbf{C}}} {\mathbf{a}}_{{\mathbf{C}}} + {\mathbf{H}}_{{\mathbf{D}}} {\mathbf{herd}}_{{\mathbf{D}}} + {\mathbf{H}}_{{\mathbf{C}}} {\mathbf{herd}}_{{\mathbf{C}}} + {\mathbf{e}}, $$

where $\mathbf{l}$ is a 37,996-vector of unobserved liabilities; ${\varvec{\upbeta}}$ is the vector of systematic fixed effects; ${\mathbf{P}\mathbf{e}}_{\mathbf{D}}$ is the vector of permanent environmental effects (random effect of identity, due to multiple records per individual) of order 8789; ${\mathbf{P}\mathbf{e}}_{\mathbf{C}}$ is the 8789-vector of random permanent environmental effects provided by the opponent; ${\mathbf{a}}_{\mathbf{D}}$ and ${\mathbf{a}}_{\mathbf{C}}$ are the vectors of direct and indirect additive genetic effects, respectively, both of length 19,707; ${\mathbf{h}\mathbf{e}\mathbf{r}\mathbf{d}}_{\mathbf{D}}$ is the vector of direct herd effects (random effect of the farming environment of the focal individual) and ${\mathbf{h}\mathbf{e}\mathbf{r}\mathbf{d}}_{\mathbf{C}}$ is the vector of herd effects provided by the opponent (random effect of the farming environment of the opponent), both of order 761; and $\mathbf{e}$ is the vector of residual effects. Furthermore, $\mathbf{X}$, ${\mathbf{W}}_{\mathbf{D}}$, ${\mathbf{W}}_{\mathbf{C}}$, ${\mathbf{Z}}_{\mathbf{D}}$, ${\mathbf{Z}}_{\mathbf{C}}$, ${\mathbf{H}}_{\mathbf{D}}$, and ${\mathbf{H}}_{\mathbf{C}}$ are the corresponding incidence matrices with the appropriate dimensions. Random effects and residuals are normally distributed with a mean of 0 and the following covariance matrix:

$$ V\left[ {\begin{array}{*{20}c} {{\mathbf{a}}_{{\mathbf{D}}} } \\ {{\mathbf{a}}_{{\mathbf{C}}} } \\ {{\mathbf{Pe}}_{{\mathbf{D}}} } \\ {{\mathbf{Pe}}_{{\mathbf{C}}} } \\ {{\mathbf{herd}}_{{\mathbf{D}}} } \\ {{\mathbf{herd}}_{{\mathbf{C}}} } \\ {\mathbf{e}} \\ \end{array} } \right] = \left[ {\begin{array}{*{20}c} {{\mathbf{A}}\sigma_{aD}^{2} } & {{\mathbf{A}}\sigma_{aDC} } & 0 & 0 & 0 & 0 & 0 \\ {} & {{\mathbf{A}}\sigma_{aC}^{2} } & 0 & 0 & 0 & 0 & 0 \\ {} & {} & {{\mathbf{I}}\sigma_{{Pe_{D} }}^{2} } & {{\mathbf{I}}\sigma_{{Pe_{D,C} }} } & 0 & 0 & 0 \\ {} & {} & {} & {{\mathbf{I}}\sigma_{{Pe_{C} }}^{2} } & 0 & 0 & 0 \\ {} & {Sym} & {} & {} & {{\mathbf{I}}\sigma_{{herd_{D} }}^{2} } & {{\mathbf{I}}\sigma_{{herd_{D,C} }} } & 0 \\ {} & {} & {} & {} & {} & {{\mathbf{I}}\sigma_{{herd_{C} }}^{2} } & 0 \\ {} & {} & {} & {} & {} & {} & {{\mathbf{I}}\sigma_{e}^{2} } \\ \end{array} } \right] \quad \left({\text{Matrix}} \;1\right)$$

where ${\sigma }_{x}^{2}$ refers to the variance of component $x$, ${\sigma }_{xz}$ is the covariance between components $x$ and $z$, $\mathbf{A}$ is the additive relationship matrix and $\mathbf{I}$ are identity matrices of appropriate size.

### Bivariate models

Bivariate (bitrait) threshold-linear animal models were run to estimate the (co)variances of social dominance with all the other traits. We built the datasets for the bivariate analyses by merging the social dominance dataset with all the other datasets, one at a time. The number of shared individuals between the social dominance dataset and each of the other datasets was, respectively, 7406 for milk production/SCS, 6318 for fertility and 6656 for morphology. Further details about all datasets are in Additional file 1. To perform bivariate analyses, we used the threshold model described above for social dominance and linear models for all the other traits. In addition to the additive genetic effect $\mathbf{a}$, random effects included in the linear models were the herd ($\mathbf{h}\mathbf{e}\mathbf{r}\mathbf{d}$) and permanent environmental effect ($\mathbf{P}\mathbf{e}$) for fertility, SCS and milk yield, the herd-test-day ($\mathbf{H}\mathbf{T}\mathbf{D}$, the effect of the farming environment and the test-day when each testing took place) [53] for SCS and milk yield and the herd-year-classifier ($\mathbf{h}\mathbf{y}\mathbf{c}$, the effect of the classifier identity in the farm and year when testing took place) for MT. Note that for simplicity, since no indirect effects are present for these traits, the different components are not indexed by D (direct effect). Referring to milk yield, SCS, fertility or any of the morphological traits as trait 1 and social dominance as trait 2, the following covariance matrices between random components (when involved) of the different traits were considered:

$$ \begin{aligned} & \left[ {\begin{array}{*{20}c} {\sigma_{a1}^{2} } & {\sigma_{a1,aD2} } & {\sigma_{a1,aC2} } \\ {} & {\sigma_{aD2}^{2} } & {\sigma_{aD2,C2} } \\ {sym} & {} & {\sigma_{aC2}^{2} } \\ \end{array} } \right] \otimes {\mathbf{A}} \quad  \left({\text{Matrix}}\; 2\right)\\ & {\text{for the genetic components}}, \end{aligned}$$

$$ \begin{aligned}&\left[ {\begin{array}{*{20}c} {{\mathbf{I}}\sigma_{Pe1}^{2} } & {{\mathbf{I}}\sigma_{{Pe1,Pe_{D2} }} } & {{\mathbf{I}}\sigma_{{Pe1,Pe_{C2} }} } \\ {} & {{\mathbf{I}}\sigma_{{Pe_{D2} }}^{2} } & {{\mathbf{I}}\sigma_{{Pe_{D2,C2} }} } \\ {sym} & {} & {{\mathbf{I}}\sigma_{{Pe_{C2} }}^{2} } \\ \end{array} } \right] \quad \left({\text{Matrix}}\; 3\right) \\ &{\text{for the permanent environmental effects}},\end{aligned}$$

$$\begin{aligned} &\left[ {\begin{array}{*{20}c} {{\mathbf{I}}\sigma_{herd1}^{2} } & {{\mathbf{I}}\sigma_{{herd1,herd_{D2} }} } & {{\mathbf{I}}\sigma_{{herd1,herd_{C2} }} } \\ {} & {{\mathbf{I}}\sigma_{{herd_{D2} }}^{2} } & {{\mathbf{I}}\sigma_{{herd_{D2,C2} }} } \\ {sym} & {} & {{\mathbf{I}}\sigma_{{herd_{C2} }}^{2} } \\ \end{array} } \right] \quad \left({\text{Matrix}}\; 4\right) \\  &{\text{for the herd effects}},\end{aligned}$$

$$ \begin{aligned} &\left[ {\begin{array}{*{20}c} {{\mathbf{I}}\sigma_{e1}^{2} } & 0 \\ 0 & {{\mathbf{I}}\sigma_{e2}^{2} } \\ \end{array} } \right] \quad \left({\text{Matrix}}\; 5\right) \end{aligned}$$

for the residuals, where $\mathbf{I}$ are identity matrices of appropriate size. The residual covariance between traits was set to zero, as different traits were not recorded at the same time. Further information on all traits and on the fixed and random effects included in their respective models are in Additional file 1 and Additional file 2: Table S1.

### Constraint of direct and indirect genetic variances and their covariance

The residual variance of the social dominance trait was set to 1 in both the single-trait and bivariate analyses as it is related to the inverse probit link function [54]. The genetic (and permanent environment) variances relative to the direct and the indirect genetic component of social dominance refer to the same phenotype, and the choice of assigning one fighter the role of ‘focal’ and to the other the role of ‘opponent’ is totally arbitrary. Thus, additive (and permanent environmental) genetic variances of the direct and indirect components should be equal, and their covariance should have the same magnitude but with a negative sign, to force the genetic correlation between direct and indirect components to be exactly − 1.

Previous research on the quantitative genetics of social dominance in Red Deer (*Cervus elaphus*) used single-trait animal models, which either estimated direct and indirect variances separately (‘no constraints’) or constrained them to be equal and the correlation to be equal to − 1 (‘constrained’) [11]. Both models were solved using penalized quasi-likelihood (as the response variable is binary) with the ASReml software [55]. Sartori et al. [12] used a Bayesian approach of Gibbs sampling (Blupf90 family of software, [56]) for a ‘no constraints’ single-trait animal model of social dominance in cattle. In our analysis, we chose to use both approaches for the single-trait analysis of social dominance. On the one hand, we believe that the ‘no constraints’ models can be used to empirically recover the values of the variances, i.e., it helps showing the limits of the actual dataset. On the other hand, the ‘constrained’ model forces these assumptions to be true and thus recovers results that are closest to the biological reality.

### Estimation of the genetic parameters

The heritability for all traits except social dominance was estimated by using only the direct components of traits estimated in the classical single-trait analysis reported above. Thus, direct heritability is the ratio between their additive genetic component ($\upsigma _{{\text{a}}}^{2}$) and the phenotypic variance $\upsigma _{{\text{p}}}^{2}$, i.e. the sum of all other variances, and it comprises the additive genetic variance, permanent environmental variance, the variance associated with other random effects when present (e.g. the ‘herd’ for milk yield, SCS and fertility, the ‘HTD’ for milk yield and SCS) and the residual variance: $\upsigma _{{\text{p}}}^{2} =\upsigma _{{\text{a}}}^{2} +\upsigma _{{{\text{pe}}}}^{2} +\upsigma _{{\text{e}}}^{2} \left( { +\upsigma _{{{\text{herd}}}}^{2} +\upsigma _{{{\text{HTD}}}}^{2} } \right)$. Heritability is thus calculated as: ${\text{h}}_{{\text{D}}}^{2} =\upsigma _{{\text{a}}}^{2} /\upsigma _{{\text{p}}}^{2} .$

For social dominance, the phenotypic variance comprises both direct and indirect components and their covariances of herd, permanent environment and additive genetic effects, that is $\upsigma _{{\text{p}}}^{2} =\upsigma _{{{\text{a}}_{{\text{D}}} }}^{2} +\upsigma _{{{\text{a}}_{{\text{C}}} }}^{2} +2\upsigma _{{{\text{a}}_{{{\text{DC}}}} }} +\, \upsigma _{{{\text{Pe}}_{{\text{D}}} }}^{2} +\upsigma _{{{\text{Pe}}_{{\text{C}}} }}^{2} +2\upsigma _{{{\text{Pe}}_{{{\text{DC}}}} }} +\upsigma _{{\text{herd}_\text{D}}}^{2}+\upsigma _{{\text{herd}_\text{C}}}^{2}+2\upsigma _{{\text{herd}_\text{DC}}}+\upsigma _{{\text{e}}}^{2}  .$ Thus, we calculated direct heritability as $\upsigma _{{{\text{a}}_{{\text{D}}} }}^{2} /\upsigma _{{\text{p}}}^{2} ;$ indirect heritability as $\upsigma _{{{\text{a}}_{{\text{C}}} }}^{2} /\upsigma _{{\text{p}}}^{2} ;$ and total heritability of the trait—including both the direct and indirect genetic components, and the covariances between them—was calculated as ${\text{h}}_{{{\text{tot}}}}^{2} = (\upsigma _{{{\text{a}}_{{\text{D}}} }}^{2} +\upsigma _{{{\text{a}}_{{\text{C}}} }}^{2} + 2\upsigma _{{{\text{a}}_{{{\text{DC}}}} }} )/\upsigma _{{\text{P}}}^{{2}}$ (see also [37]). Note that in the ‘constrained’ analysis, only one parameter is calculated for the additive genetic effect $\left( {\upsigma _{{\text{a}}}^{2} } \right)$ and one for the permanent environmental variance $\left( {\upsigma _{{{\text{Pe}}}}^{2} } \right)$. This parameter is equivalent to either the direct or indirect variance in the unconstrained analysis, and thus, to obtain a total heritability of 0, in the constrained analysis, we have $\upsigma _{{\text{P}}}^{2} =\upsigma _{{\text{a}}}^{2} +\upsigma _{{\text{a}}}^{2} - \left( {2\upsigma _{{\text{a}}}^{2}} \right) +\upsigma _{{{\text{Pe}}}}^{2} +\upsigma _{{{\text{Pe}}}}^{2} - \left( {2\upsigma _{{{\text{Pe}}}}^{2}} \right) + \upsigma _{{\text{e}}}^{2} = 1.$ This happens because by constraint there is only one parameter estimated for the additive and one for the permanent environmental (co)variances (however covariances have to be multiplied by − 1). Thus, both $\upsigma _{{\text{a}}}^{2}+\upsigma _{{\text{a}}}^{2} - \left(2 {\upsigma _{{\text{a}}}^{2}} \right)\;{\text{and}}\;\upsigma _{{{\text{Pe}}}}^{2} +\upsigma _{{{\text{Pe}}}}^{2} - \left( 2{\upsigma _{{{\text{Pe}}}}^{2}} \right)$ are equal to 0, and consequently the phenotypic variance is given by the residual variance only, which in turn is equal to 1 $\left( {\upsigma _{{\text{P}}}^{2} =\upsigma _{{\text{e}}}^{2} = 1} \right)$, because $\upsigma _{{\text{e}}}^{2}$ in categorical traits is fixed to 1, and it is thus possible to calculate direct and indirect heritability as $\upsigma _{{\text{a}}}^{2} /1.$

The genetic correlations (${r}_{a}$) between the direct components of each pair of traits were calculated as ${r}_{a}={\upsigma }_{a1,aD2}{{/(\upsigma }_{a1}^{2}\times{\upsigma }_{aD2}^{2})}^{0.5}$; the genetic correlations between direct and indirect genetic components of social dominance were calculated as ${\upsigma }_{aD2,C2}/{{(\upsigma }_{aD2}^{2}{\times\upsigma }_{aC2}^{2})}^{0.5}$; and the genetic correlations between the direct component of trait 1 and indirect social dominance were calculated as ${\upsigma }_{a1,aC2}/{({\upsigma }_{a1}^{2}\times{\upsigma }_{aC2}^{2})}^{0.5}$.

All ‘no constraint’ analyses were run via Bayesian inference, applying the Gibbs sampling algorithm with flat priors and using the program THRGIBBS3f90 of the BLUPF90 package [56]. All the analyses considered 600,000 iterations of the Gibbs sampler, with a starting burn–in that discarded 100,000 iterations. The posterior mean of 5000 samples (one every 100 samples) was considered as parameter estimator, and the lower and upper bounds of the 95% highest posterior probability density regions (HPD95) were used as estimation errors. Estimates were considered significant when 0 was not included in the HPD95. Posterior distribution analyses were performed using the POSTGIBBSF90 program [56]. The convergences of the Gibbs sampling chains were checked by visual inspection. While THRGIBBS3f90 was our software of choice because of its precision, speed, and specialization in handling complex models and livestock dataset [56], this software does not permit to constrain variances to be equal, neither in single—nor in bivariate analyses. Because of our need for repeated iterations to estimate EBV, and of the complexity of our models, we decided not to use the AIREML-derived algorithms [57] and thus, to try the ‘constrained’ version of our model, we used the MCMCglmm package [58] for R [59], which fits generalised linear mixed models using Markov chain Monte Carlo techniques. Within MCMCglmm, we implemented the constraint ${|\upsigma }_{{\text{a}}_{\text{D}}}^{2}| = {|\upsigma }_{{\text{a}}_{\text{C}}}^{2}| = |{\upsigma }_{{\text{a}}_{\text{DC}}}|$ in a single-trait animal model analysis of social dominance using the multimembership model formulation ‘mm (focal—opponent)’. This formulation estimates only one variance parameter (${\upsigma }_{{\text{a}}_{\text{D}}}^{2}$ or ${\upsigma }_{{\text{a}}_{\text{C}}}^{2}$, the distinction is not important) for social dominance, which de facto forces ${\upsigma }_{{\text{a}}_{\text{D}}}^{2}$ = ${\upsigma }_{{\text{a}}_{\text{C}}}^{2}$  = − ${\upsigma }_{{\text{a}}_{\text{DC}}}$. Unfortunately, currently MCMCglmm does not allow to implement a bivariate analysis between a trait with a ‘mm (focal—opponent)’ specification and another trait with only the animal effect (J. Hadfield, pers. communication), which made it impossible to apply this constraint also to our bivariate analysis. Moreover, MCMCglmm tends to be slow with pedigrees such as those commonly used in animal breeding, which contain inbreeding loops; therefore, to run it, we had to use a subset of our dataset and completely remove the random effect of herd, otherwise it would not converge. We ran our single-trait constrained model with 350,000 iterations, checking the convergence of chains visually. Effective chain size was always larger than 2335 for fixed effects and 1602 for random effects.

We obtained individual EBV (or the individual heritable part of a trait) as solutions of our THRGIBBS1F90 single-trait estimates for the direct genetic effect of fertility, milk yield, SCS, morphological traits, and direct/indirect genetic effect of social dominance. We assessed annual genetic trends by plotting the evolution over time of the average EBV of individuals born in the same year. The time window from 2000 to 2015 was considered to include years with at least 100 newborns. To further test the presence of evolutionary change for the specific traits within the population, we also adopted the protocol described in Hadfield et al. [60] to calculate the probability that the estimated rate of change might be different from the null model of evolutionary change by drift alone.

## Results

### Single-trait models and heritability estimates

The variance components for all the traits, which were estimated using single-trait animal models, are reported in Additional file 2: Table S2. The corresponding heritability estimates expressed as posterior means with corresponding 95% HPD are in Table 1. Genetic parameters for social dominance were expressed on a liability scale, since it is a binary trait. In the analysis of social dominance without constraints, the direct component of the trait had a heritability of 0.1258 [HPD95 = 0.0957; 0.1607], which is similar to that of the indirect component, i.e. 0.1213 [0.0909; 0.1557]. The total heritability for social dominance was close to 0, i.e., 0.0034 [0.0003; 0.0145], and the coefficient of genetic correlation between direct and indirect genetic components was − 0.9887 [− 1.0000; − 0.9437]. In the constrained model of social dominance, which was run using the MCMCglmm package, a heritability of 0.1646 [0.1320; 0.2003] was found, after adding the influence of fixed effects using the procedure described in de Villemereuil et al. [61, 62]. Note that when we used our full dataset and the pedigree information, it took the program approximately seven days to run 30,000 iterations: thus, we used subsets of different sizes to run our analyses which led to nearly identical results regardless of the subset used.

**Table 1:** Heritability of traits

Trait	*h*^2^(± SE)	CI^−^	CI^+^
Social dominance (constrained, without herd effect)	0.1646 (± 0.0130)	0.1320	0.2003
Direct social dominance	0.1258 (± 0.0166)	0.0957	0.1607
Social dominance total	0.0035 (± 0.0039)	0.0003	0.0145
Indirect social dominance	0.1213 (± 0.0164)	0.0909	0.1557
Milk yield	0.2244 (± 0.0092)	0.2062	0.2423
Somatic cells score	0.0602 (± 0.0051)	0.0506	0.0705
Fertility	0.0251 (± 0.0043)	0.0170	0.0339
Morphological traits			
Fore udder attach	0.1535 (± 0.0112)	0.1320	0.1754
Udder overall	0.1518 (± 0.0110)	0.1308	0.1736
Rear udder attach	0.1413 (± 0.0110)	0.1197	0.1632
Udder width	0.1389 (± 0.0112)	0.1168	0.1613
Thinness	0.0754 (± 0.0093)	0.0581	0.0942
Front muscularity	0.1888 (± 0.0116)	0.1663	0.2117
Thorax depth	0.2116 (± 0.0121)	0.1882	0.2356

Heritability of traits is expressed as a mean and its standard error (SE), and the confidence interval (CI) expressed as of the 95% high posterior density interval of Gibbs sampling estimates (95% high posterior density interval of MCMC sampling estimates for the MCMCglmm software). All estimates were obtained by running single-trait analysis. Except for social dominance that has a direct, indirect and total heritability, the other traits only have a (direct) heritability

Except for social dominance, that included an indirect component, the heritability for all the other traits was computed using only direct components. The estimates of heritability for cow fertility, somatic cell score and milk yield were equal to 0.025 [0.017; 0.034]), 0.060 [0.051; 0.071], and 0.224 [0.206; 0.242], respectively. The estimates of heritability for the morphological traits ranged from low for thinness (0.075 [0.058; 0.094]), to moderate for traits linked with udder size and morphology (0.139 ≤ h^2^ ≤ 0.1535) and high for front muscularity (0.189 [0.166; 0.212]) and thorax depth (0.212 [0.188; 0.236]).

### Bivariate models and correlations

The genetic and permanent environmental covariances were estimated using a bivariate animal model and are reported in Additional file 2: Table S3. The genetic correlations (${r}_{a}$) of social dominance (both direct and indirect components) with each of the other traits are in Table 2. The estimates of heritability for all the target traits obtained by using the bivariate models or the single-trait models were similar (see Additional file 2: Table S4). Note that, since we could not constrain social dominance variance components (direct and indirect) in the bivariate analyses, correlations are present twice for each of our bivariate analyses. However, differences in the correlations do not have a biological meaning, but are by-products of using empirically estimated (co)variances from a real-life dataset.

**Table 2:** Genetic correlations (*r*_*a*_) between social dominance vs. the other traits

Trait	*r*_*a*_ of trait with direct dominance			*r*_*a*_ of trait with indirect dominance		
	Mean ± (SE)	CI^−^	CI^+^	Mean ± (SE)	CI^−^	CI^+^
Milk yield	− *0.1934* (± 0.0604)	− 0.3078	− 0.0726	*0.1341* (± 0.0602)	0.0172	0.2497
Somatic cell score	*0.3401* (± 0.0690)	0.2080	0.4738	− *0.1790* (± 0.0831)	− 0.3503	− 0.0234
Fertility	*0.3692* (± 0.0945)	0.1760	0.5488	− *0.4690* (± 0.0822)	− 0.6228	− 0.3026
Morphological traits						
Fore udder attach	− 0.0036 (± 0.0686)	− 0.1407	0.1246	− 0.0160 (± 0.0651)	− 0.1428	0.1114
Udder overall	0.0051 (± 0.0729)	− 0.1469	0.1415	− 0.0623 (± 0.0681)	− 0.1946	0.0758
Rear udder attach	− 0.1035 (± 0.0734)	− 0.2470	0.0415	0.0269 (± 0.0709)	− 0.1147	0.1634
Udder width	− 0.0848 (± 0.0745)	− 0.2335	0.0567	0.0152 (± 0.0713)	− 0.1246	0.1576
Thinness	− 0.0329 (± 0.0862)	− 0.2010	0.1362	− 0.0424 (± 0.0855)	− 0.2106	0.1238
Front muscularity	*0.2660* (± 0.0665)	0.1377	0.4267	− *0.3002* (± 0.0654)	− 0.4267	− 0.1723
Thorax depth	*0.2485* (± 0.0660)	0.1226	0.3847	− *0.3250* (± 0.0610)	− 0.4403	− 0.2008

Genetic correlations (*r*_*a*_) obtained by running bivariate analyses for each trait pair including social dominance vs. the other traits. The posterior means of the Gibbs samples have been reported with the respective standard errors (SE) and the 95% high posterior density confidence interval (CI) of Gibbs sampling estimates. When CI did not contain zero, the mean is in italics

Social dominance showed a strong genetic correlation with fertility (respectively ${r}_{a}$ = 0.369 [0.176; 0.549] for the direct genetic component and ${r}_{a}$ = − 0.469 [− 0.623; − 0.303] for the indirect genetic component). Given that fertility is measured as ‘months of interval between parturition and conception’, higher values correspond to lower fertility. Thus, the more dominant an individual is (high values for the direct component of social dominance), the lower is its fertility, while the less dominant an individual is—and therefore more subordinate—the higher is its fertility. High SCS values indicate a larger number of somatic cells in milk after an inflammation of the mammary gland, such as mastitis, and therefore lower udder health. SCS was genetically correlated with social dominance (${r}_{a}$ = 0.340 [0.208; 0.474] for the direct component and ${r}_{a}$ = − 0.179 [− 0.350; − 0.023] for the indirect component). This means that more dominant individuals showed greater inflammation of the mammary glands. Milk yield was also genetically correlated with social dominance (direct component of social dominance ${r}_{a}$ = − 0.193 [− 0.308; − 0.073]; indirect component (${r}_{a}$ = 0.134 [0.017; 0.250]). Thus, cows that won more battles produced less milk.

The genetic correlations of social dominance with the morphological traits depended on the trait (Table 2). We found social dominance to be genetically correlated with two traits associated with more muscular and ‘male-like’ morphology, front muscularity (direct component of social dominance ${r}_{a}$ = 0.266 [0.138; 0.427]; indirect component (${r}_{a}$ = − 0.300 [− 0.427; − 0.172]) and thorax depth (direct component of social dominance ${r}_{a}$ = 0.249 [0.123; 0.385]; indirect component (${r}_{a}$ = − 0.325 [− 0.440; − 0.201]). All other correlations with morphological traits were not significant (0 was within the confidence interval, Table 2).

### Genetic trends

**Fig. 1:**
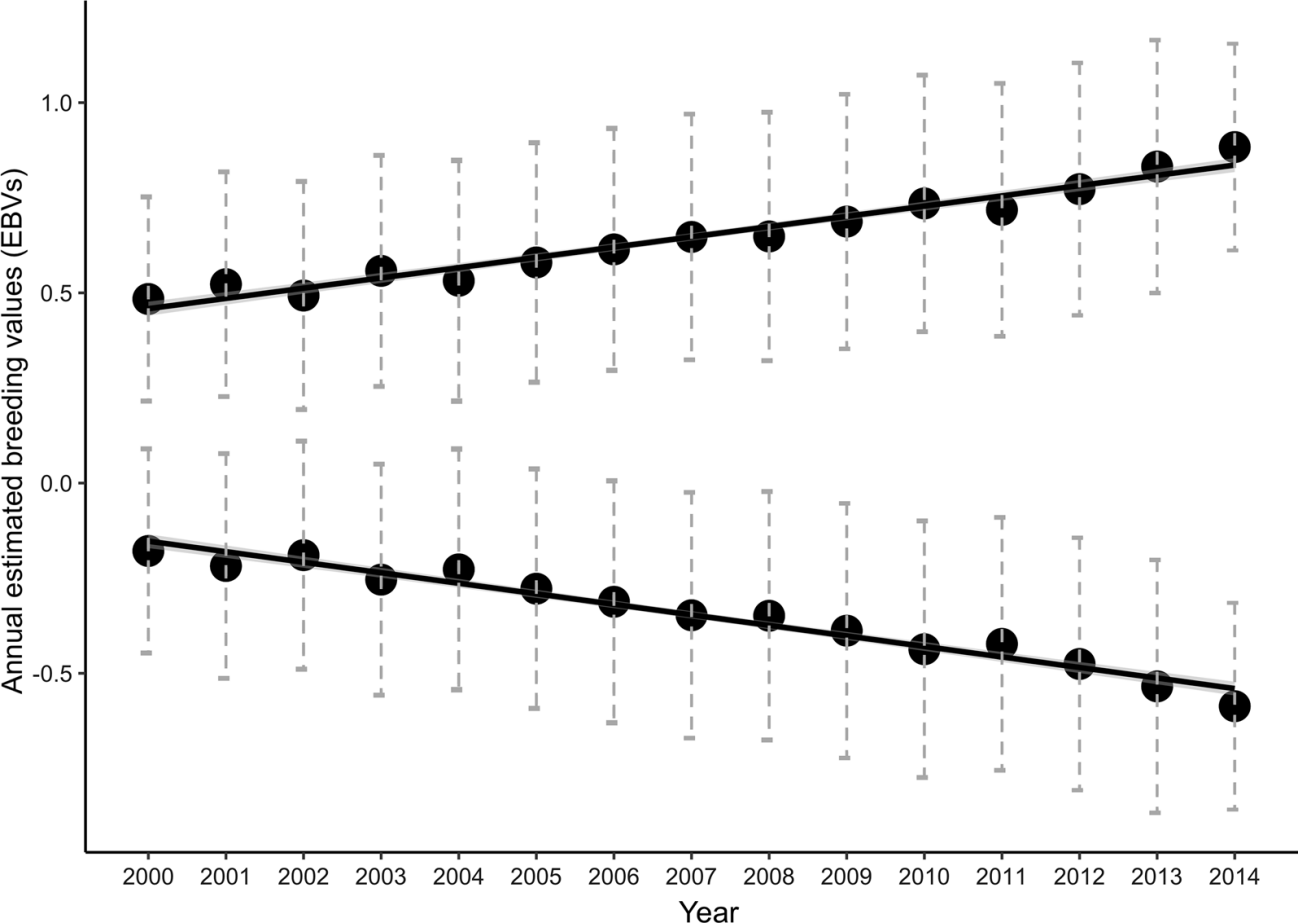
Genetic trends for the direct and indirect components of social dominance. Genetic trend for the direct and indirect genetic components of social dominance from 2000 and 2014. The average annual estimated breeding values (EBV) and standard error of the mean are represented. There was an increase in the EBV for the direct component of dominance, and an opposite decrease for the indirect component

**Fig. 2:**
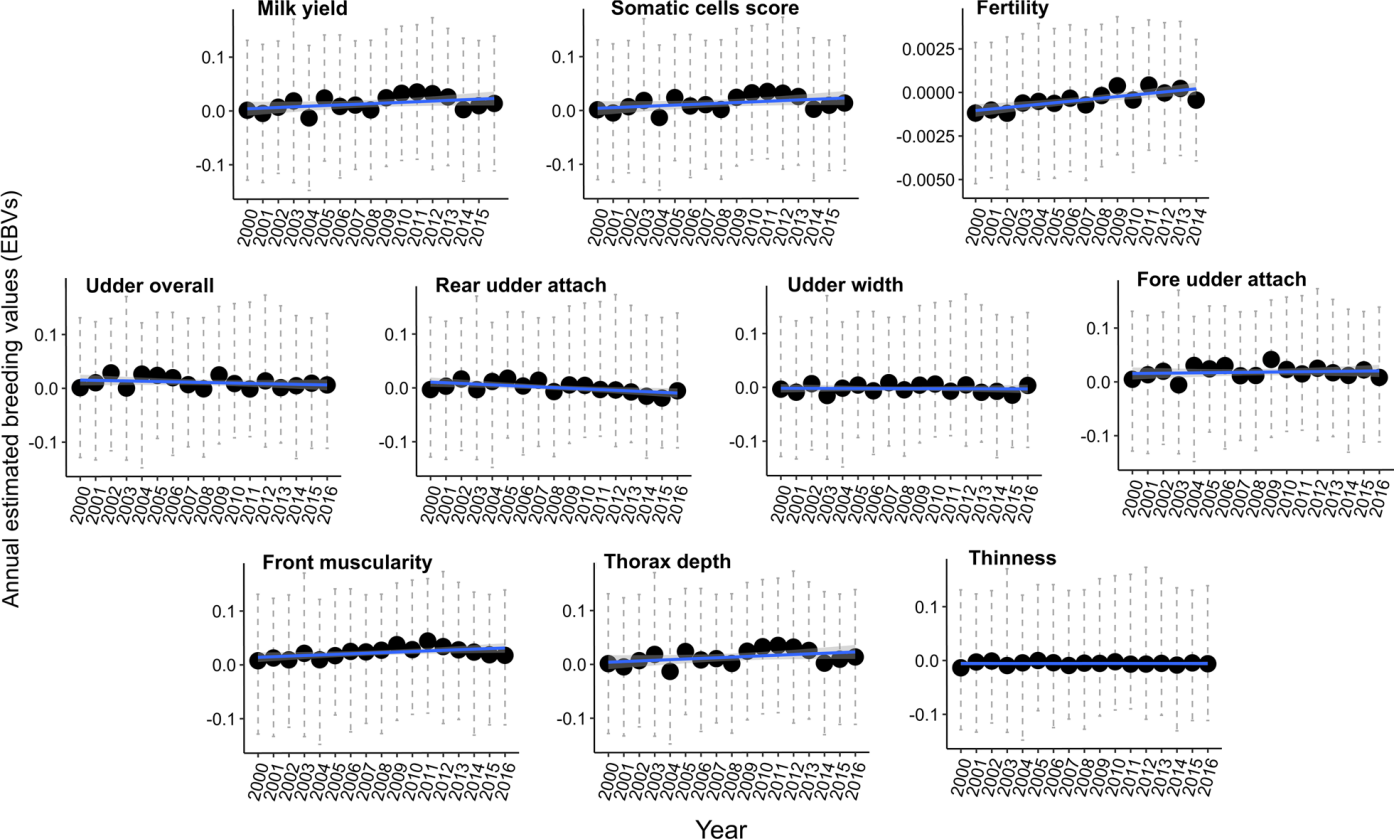
Genetic trends for life history and morphology traits. Genetic trends for life history and morphology traits from 2000 to 2014–2016 (depending on the trait). The average annual estimated breeding values (EBV) and standard error of the mean are represented

We found considerable increases in the EBV of individuals born during the 16-year period from 2000 to 2015 for the direct component of social dominance, while an opposite decrease was found for the indirect genetic component (Fig. 1) and (see Additional file 2: Table S5). Regarding the other traits, the trends varied in strength and direction (Fig. 2) and (see Additional file 2: Table S5), and to test whether they indicated a change in the EBV of the population, we calculated the percentage of MCMC replicates with regression coefficients that did not overlap with those randomly expected by drift (null model of evolutionary change). A percentage of 100% was found for the direct component of social dominance, and naturally for the indirect component all the replicates had negative regression coefficients (see Additional file 2: Table S5 and Additional file 3: Figures S1 and S2). The change in EBV showed a clear positive trend for three traits, with a high percentage of replicates whose regression coefficients were higher than those consistent with the null model (SCS 95.3%, front muscularity 99.4%, thorax depth 99.8%). All other traits had trends that did not consistently differ from the null model (from 33.9% for udder overall to 62.1% for fore udder attach): all the distributions are reported in Additional file 2: Table S5 and in Additional file 3: Figures S3–12.

## Discussion

Our results provide evidence that selection for dominant individuals in a population can contribute to the genetic evolution of other traits via their genetic correlations with social dominance. We obtained this result by modelling social dominance as a phenotype and estimating both its direct and indirect genetic effects, and for the first time, we detected significant genetic correlations of both components with fitness, health, and morphological traits. As expected, our bivariate models showed that social dominance is a heritable trait, which is consistent with the heritability of other social behaviours [63] that have been reported for domestic [17] and wild populations [64, 65].

We found antagonistic genetic correlations between winning agonistic interactions and traits associated with cows’ health and fitness. Cows that won more fights had on average a lower fertility, decreased milk yield and poorer udder health [34, 35]. The most dominant cows had also ‘male-like’ morphological features, such as a bulkier body in terms of thorax depth and front muscularity. As expected, the genetic correlations of the direct and indirect components of social dominance with the other traits were symmetrical, i.e. of the same magnitude but with opposite signs. These results are important for two reasons: first, they show how social behavioural traits, such as social dominance, are genetically correlated with morphological, health and life-history traits. Second, our results suggest that selection for social dominance is not only associated with a clear evolutionary response in this trait, but that this evolutionary response is also ‘dragging’ along some correlated traits. In fact social dominance showed a strong trend of change in its EBV, with the direct component showing a marked increase and, of course, the indirect component showing a mirroring decrease. This means that, while the phenotype cannot evolve, the genetic merit for social dominance of individuals born more recently is markedly greater than that of their ancestors. In other words, cows in our population have evolved an increased fighting ability in response to selection for social dominance; but it is impossible to observe this at the phenotypic level, because the mean of a 1/0 trait cannot change. However, the traits correlated with social dominance can change: we found that morphological features that are linked with greater frontal muscularity had increased EBV, while the EBV for mammary health decreased. These traits changed according to their correlation with the target of selection, i.e., social dominance. In 2012, selection breeding programs in Aosta cattle introduced weights of 50% for milk yield, 40% for social dominance and 10% for meat yield, but the main unofficial target trait under selection has always been social dominance in this breed, with milk and meat yields being of very little importance in the selection choices [17, 66, 67]. Although we cannot state that the observed genetic trends are only due to the selective pressure for social dominance, we argue that it was the most important factor that drove the evolutionary change of the correlated traits. Higher genetic merit for social dominance, given the antagonistic correlation with milk yield, is not likely to be a consequence of selection for milk yield, and the ‘meat’ component is by far the lowest trait in the index (10%). While selection for milk yield might have played a role in the trend observed for SCS, it is very unlikely that it is responsible for the trends that we observed for morphological features. Indeed, this is because the trend of change observed for muscularity is opposite to what would be expected if only milk yield was included in the selection index, which would lead to a more ‘female-like’ body conformation spreading in the population. Thus, our results provide strong evidence that social dominance as a behavioural trait can not only evolve, but can also impact the evolution of other traits.

### Variance constraints in dyadic contest outcomes

A social phenotype such as dominance can only be modelled by considering at the same time both the variance attributed to the genetic makeup of the focal (direct) and the variance attributed to the genetic makeup of the opponent (indirect) individuals. Since the phenotype associated with each fight is the same for both the focal and opponent individuals, variances are expected to be equal, and their covariance to have the same magnitude but with opposite signs. On the one hand, certain software or packages (e.g., MCMCglmm) can add this constraint to the animal model, which ensures that the correlation between direct and indirect components is − 1 and the total heritability for social dominance is 0. On the other hand, an unconstrained model can test the size and completeness of the dataset, since the values that it estimates freely approximate these features. Our unconstrained single-trait model obtained a correlation of − 0.989 between direct and indirect components of social dominance and a total heritability of 0.003. These very close approximations to − 1 and 0 were also obtained in all subsequent bivariate analysis (see Additional file 2: Table S4). The heritability estimates for the direct and indirect components were 0.126 and 0.121, respectively, not so different from that obtained by the constrained model (0.164). The difference in the estimates of heritability between the constrained and the unconstrained models is probably due to the fact that, in the constrained model, we could not insert herd as a random effect, neither for the focal nor for the opponent individual, since it would have impeded chain convergence. In our bivariate analysis, the slight differences in (co)variances between direct and indirect effects do not carry any biological meaning and are likely just an artefact that is caused by the use of a real-life dataset. Taken together, we believe that our unconstrained analysis was more than sufficiently powerful to recover realistic estimates, as it always approximated very closely both the features of the social dominance model and the heritability obtained with the constrained single-trait model. However, it must be noted that our constrained model also produced good estimates despite running only on a small sub-sample of the dataset, which indicates that a constraint might help save power and accuracy when the dataset is small. Finally, differences between the direct and indirect correlations do not have biological meaning, and the most realistic estimate of their magnitude, when they differ, would probably be an average of the two posterior distributions.

### Features and limitations of our dataset

It is very difficult to estimate the IGE of behaviour [65, 68] and collecting a dataset of sufficient size and with enough details to estimate the genetic correlations of IGE with other traits is even more complicated [69]. The unique opportunity given by the meticulously recorded ‘Batailles des Reines’ in the Aosta Chestnut-Black Pied cattle breed created the conditions for a decade-long study with tens of thousands of individuals. However, the dataset did have some limitations: for example, we could not model dominance in male individuals to check for sexually antagonistic pleiotropy in the investigated traits, since the males do not participate in the contests. Nonetheless, the artificial nature of the agonistic interactions allowed us to avoid several of the issues often encountered when modelling quantitative genetic data in a natural population [70, 71]. Studies on the genetics of social behaviours in natural populations struggle to partition the variance that is linked to the social environment [2, 65]. Nepotism, brood effects, and the presence of other related individuals tend to inflate the variance that is attributed to genetic effects, especially in small populations [4, 18, 72] and (Susan Alberts, personal communication). Modelling these factors through IGE is often much too complex, given that the nature of the interactions is often communal, highly sparse, and discontinuous [65]. Given how agonistic interactions are structured in the ‘Batailles des Reines’ (see Methods), we could discard issues that are linked with the immediate social environment, such as possible interference of other conspecifics with the two individuals engaged in an ongoing duel (e.g., [73]). In addition, the repeated nature of these battles (i.e., individuals may compete more than once within an annual tournament and over the years) permitted a randomized and standardized evaluation of social dominance. This also allowed us to partition the trait variance relative to the (non-genetic) individual permanent environment, an achievement that is rarely possible in most studies under natural conditions [72]. Two additional specific features of our system are worth mentioning, as they might have partially influenced our results. The first is that cows are chosen to fight within weight classes. In other words, two cows that greatly differ in weight might never face each other in tournaments. We do not have direct evidence that size directly influences social dominance in Aosta cows, but it does in many wild [74] and livestock [75] species, with larger animals being usually more dominant [24]. Thus, we could expect that if some cows were to fight adversaries outside their weight class, it could influence genetic variances and correlations. Since size usually has a large genetic component, a positive correlation between this component and social dominance would entail that our reported genetic variance for the latter might be underestimated. In other words, if there had been no division into weight classes, a greater part of the fights would have been decided by a factor that is strongly controlled by the genetic background of the cows. Moreover, our results show that winning cows have greater thorax depth and front muscularity, which in turn are possibly larger in cows of the ‘heavy’ fight category. This suggests that the correlation that we found between social dominance and this male-like morphological trait is also an underestimation, as across weight categories the relation between male-like appearance and winning might be more pronounced. However, with the current dataset we cannot speculate any further, since morphological traits are measured only once over a cow’s lifetime, while cows usually fight over several years, with their weight (and thus weight category) changing with age. A second factor which may have an influence on our results and is difficult to disentangle is that dominant cows have access to more fights. This raises two issues, the first one being that winning individuals might be repeated more times in the dataset. However, given the tournament-like arrangement of the ‘Batailles’, only a handful of cows out of hundreds get to fight more than 1 to 3 times in one day, thus any effect due to this issue is likely very small. The second issue is a possible winner effect: since cows that win more fights proceed further into the tournament, there might be a positive effect of previous wins—but, as with most repeated measures of social dominance, it would be difficult to disentangle an eventual winner effect from actual genetic quality. Moreover, in the set-up of our study, this is controlled by the fact that only cows that won an identical number of fights can face each other during the tournament, thus ensuring that an eventual winner effect is symmetrical in all fights.

### Generalization of our results

In general, our set-up was thus more akin to an experiment under controlled conditions than to observations of a natural situation, and, accordingly, it permitted an unprecedented degree of focus not only on the genetic basis of behavioural traits, but also on their correlation with life-history traits. In natural populations, dominant individuals usually obtain more resources and mating opportunities: differences in individual quality [76] and resource acquisition [21, 23] combine with social feedback effects [24] to create the conditions for an increased overall fitness and health of dominant individuals [77]. In other words, possible antagonistic genetic correlations between social dominance and fitness or health traits in wild populations are more than compensated by the increased gains that social dominance allows. However, in a managed population, access to resources is not limited, which eliminates the confounding factor of differences in acquisition. Thus, it is possibly for this reason that, in our study, we found antagonistic genetic correlations between social dominance and fitness traits that are more rarely found in natural populations [78].

### Genetic correlation between social dominance and fertility

The genetic correlation between social dominance and fertility is especially interesting from an evolutionary point of view. Fertility is a key fitness trait and, as with most life-history traits, its deviations from the population optimum are thought to be under strong negative selection [79]. In our study, fertility had a heritability estimate of ~ 0.025, in line with previous studies [34, 49] and thus, it was surprising to find that it presented strong antagonistic genetic correlations with social dominance. The genetic constraints and ontogeny of these traits appear to be robustly linked, possibly through hormonal pleiotropy [80], as masculinizing hormones, such as testosterone, are often linked to better competitive performances [81]. However, these hormones can also directly affect female fertility through the modification of primary sexual traits [32]. In our study, we found that the breeding value of fertility does not decrease over time, despite the selection of dominant individuals in this population. Given the low heritability of fertility, the slope associated with the change in the EBV of the cohort was higher than that of evolutionary change of the null model only in 52.6% of the MCMC replicates, which means that in spite of the unfavourable correlation, the rate of change is minimal; thus, it is unlikely that the trait will show a phenotypic shift in the near future [82]. However, given that fertility is a key trait for the livestock sector, this result highlights the importance of accounting for the risk that selection for social dominance (targeted or accidental) might have an impact on economically important traits linked with production and health.

### Genetic correlation between social dominance and SCS, milk yield

Social dominance also showed an antagonistic genetic correlation with SCS. Dominant individuals had a larger number of somatic cells in milk, which means poorer udder health. SCS has widely been used in animal breeding as a proxy for mastitis risk and overall udder health [51]: a higher SCS has additionally been linked with a deteriorated immune system [83]. In particular, immune responses may be expected to have antagonistic relationships with social dominance [84], as the testosterone that often mediates fighting behaviour can profoundly impact them [85, 86]. Interestingly, we found that the EBV of this trait showed significant signs of degradation, which indicates a potential undesired consequence of the selection for fighting ability. This might be a cause of concern for the future, as SCS is an indicator of great economic value [50]. However, it must also be noted that degradation of SCS might not be caused only by selection for social dominance, as SCS has also an antagonistic correlation with milk yield [50]. Although milk yield is historically less important in the selection programs of Aosta cattle [66], it is a selection target and thus could indeed play a role in the degradation of SCS.

Milk yield showed a lower, but still significant, antagonistic genetic correlation with social dominance: dominant individuals have a decreased ability to provide large quantities of milk. The production of milk is a demanding task, requiring a lot of energy and nutrients. It involves a vast mobilization of resources, which can be greatly increased by direct artificial selection for milk production. For all these reasons, it is not unexpected that it shows an antagonistic correlation with social dominance. However, since milk production is also a target of selection, the effects of the unfavourable correlation with social dominance may be diluted by the weight that is attributed to milk production in the selection index, and for this reason, we do not see a deterioration in milk production EBV. On the other hand, usually cattle that are selected only for milk production show a change in their morphology, as udder size increases, frontal muscularity decreases, and cows assume a more ‘female-like’ body conformation. This is the opposite of what is observed in the Aosta breed, since there seems to be no change in udder morphology, and on the contrary, selection for winners has led to a masculinization of the cows’ body shape.

### Genetic correlations between social dominance and morphology traits

We did not find a significant genetic correlation between social dominance and either the morphology of the udder or thinness. Although udder conformation traits are indicators of a ‘female-like’ conformation and would have been expected to trade-off with dominance related traits [32], in our population, udder morphology does not appear to show a clear trend of change, neither negative nor positive, which could be a consequence of the dual selection for both social dominance and milk production. However, front muscularity and thorax depth, two traits that evaluate key aspects of the bulky, wiry and ‘male-like’ appearance of cows, were not only genetically correlated with social dominance, indicating that more ‘male-like’ individuals won more agonistic interactions, but also showed changes in this population, as illustrated by our results. The change in the cows’ appearance over the years is the kind of fast evolutionary response that is typical of artificial selection. Indeed, while research has made a lot of progress on the neural pathways and physiology of aggressiveness and social dominance, it has been more difficult to quantify how selection for social dominance would change the shape of an animal body. Our results show that, as dominant individuals are selected for, the morphology of the population evolves. This evidence of a link between skeletal and muscular development with social dominance is significant because, while the very specific morphological traits used in our study did not have a high heritability, morphology is often associated with greater heritable variation and response to selection than behavioural and life-history traits [87]. Thus, a genetic correlation between morphological traits and social dominance becomes especially important in terms of genetic variability, selection, and potential evolution within social groups; indeed, we show that a population under strong selection for dominance could in fact greatly and rapidly change its morphology over time. Finally, our results have exciting implications for the genetic architecture of social dominance. A lot of work has been done on the genetic architecture of muscularity in cattle [88, 89] and on the genetic and molecular basis of social dominance behaviour in various species [90, 91]. The Aosta breed is a very good candidate to investigate what are the functional elements, genes and pathways that might be common to both traits, and that are thus at the basis not only of the genetic correlation, but also of the common evolutionary trajectory of the two traits.

### Social dominance and evolution

Indeed, a key conclusion that can be derived from our study is that selection for social dominance has the evolutionary potential to affect other traits with very diverse genetic architectures [92]. Several of the traits that we considered here are, in fact, linked by antagonistic relationships of their own: for example, mastitis risk is often positively correlated with milk production [53], which in turn is negatively correlated with fertility [93]. Social dominance is, thus, linked to entirely different aspects of the cows’ reproductive biology, which implies the presence of several interconnected mechanisms of pleiotropy [94]. Indeed, in spite of the categorical nature of its phenotypic record, the expression of social dominance involves neural, hormonal, and physiological responses that are regulated by a myriad of factors, and thus, it is very difficult to piece together what are exactly the genomic targets of selection in this trait. The layered genetic architecture of this trait has far-reaching consequences: since social dominance is the most important factor in the selection of the Aosta Chestnut-Black Pied breed, artificial selection of dominant individuals is shown to affect not only the evolution of their behaviour, but also all the physiological and morphological traits that are pleiotropically linked with it. Through several interconnected pathways, the antagonistic genetic correlations with female-like and health-related traits might lead in the long term to a decrease of the average health of the population. The EBV of SCS in our dataset showed a significant degradation of the trait, present in 95.3% of all MCMC replicates. While there is a lag dividing this observation from actual phenotypic change [82], especially given that Aosta cows generally show good phenotypic values for fertility and SCS in comparison with cosmopolitan and intensively selected breeds, if the current selection trend continues, it is conceivable that key life history and health traits could worsen in the population. This is consistent with what has been reported for the Hérens breed, which was historically selected for fighting ability, and tends to show low values for fertility and milk yield [95]. In natural populations, where competition for resources is strong, covariance of life-history traits with social dominance could therefore lead them away from the optimum, causing local maladaptation [96]. Moreover, since life history, fitness, and health traits correlate not only with the direct but also with the indirect component of social traits, their rate of evolutionary change might be even faster [5]. For example, in populations of highly sociable species, better opportunities for dominant individuals might be directly linked with worse opportunities for the subordinates, particularly where resources are scarcer [97], depending on the context [98]. Finally, another major consequence of the selection of covarying traits might be the evolution of the correlations themselves. For example, varying degrees of the stringent endocrine constraint between fertility and social dominance might be differently advantageous and thus, in time, change their frequencies in the population [99]. Future studies should address whether genetic correlations between different traits might themselves be under selection and thus evolving in this and other populations.

Finally, social dominance represents a specific case of IGE, i.e. a competitive model where the effect of the opponent is key to obtaining realistic estimates of heritability for the trait but perfectly mirrors the effect of the focal individual. However, this might not be the case with other traits and situations. For example, our study did not consider any possible IGE that acts directly on fertility, milk production, morphology, etc. In fact, it is possible that these traits are themselves influenced by the diverse genotypes of their groupmates, especially if housed in small groups during their development. Indeed, extensive work has shown that, for example, aggressive behaviour in pigs is linked to decreased groupmates’ weights [100] and that several health traits are linked to significant social effects [101]. In Aosta cows, IGE that act directly on these traits are expected to be more diluted, as they are never housed in small or barren pens and are free to roam during summer. Thus, it was not possible to add this herd-mates effect to our study: in fact, within the matrices shown [Matrices (2) to (4)], we did not include the potential IGE acting on trait 1 (fertility, SCS, morphology, etc.). However, in future studies, provided an adequate dataset is available, it should be possible to perform bivariate analyses of two traits each with their own IGE. Moreover, besides for social dominance, IGE could be essential for the study of several other traits with values that are in part, or completely, dependent on the values of other individuals, such as, for example, leader and follower dynamics [102], or group success [1] where the value of a trait is the same for the entire group.

## Conclusions

The potential value of studies on the genetic correlation between social dominance (modelled with IGE) and life history and fitness traits has been discussed for several years. Our work uncovers evidence of a genetic correlation between a behavioural trait (social dominance) and key life-history, fitness, and morphological traits. We show that dominance interactions, such as those that are ubiquitous within animal groups, are genetically correlated, via both direct and indirect components, to other traits. Artificial selection for social dominance in our population plays a significant role in the evolution of these traits, by reducing the cows’ reproductive efficiency and increasing morphological masculinization. We argue that social dominance in livestock can be a useful model to investigate the genetics of social behaviour and other social traits.

### Supplementary Information

**Additional file 1. **In-depth description of the single-trait models for milk yield, somatic cell score, fertility and morphological traits [34, 49–53, 103, 104].

**Additional file 2: Table S1.** Descriptive statistics of the traits, including the extreme phenotypic values, the phenotypic mean and its standard deviation (SD). **Table S2.** Variance components of the traits expressed as mean ± standard error (SE) of posterior density intervals of Gibbs sampling estimates, obtained by running single-trait analysis. **Table S3.** Genetic (σ_a1a2_) and permanent environmental covariances (σ_pe1pe2_), obtained by running bivariate analyses for each pair of traits including social dominance vs. the other traits. **Table S4.** Heritability estimates for the traits of interest obtained with bivariate analyses. Posterior means for heritability estimates with their respective standard error (SE) and the 95% high posterior density confidence interval (CI) of Gibbs sampling estimates obtained for the traits of interest by running bivariate analyses. **Table S5.** Regression coefficients (slopes) of the trait variations over time calculated using the average estimated breeding values (EBV) for the newborns of target years (genetic trends) and % of replicates with slope > 0.

**Additional file 3: Figure S1.** Distribution of the regression coefficients (slopes) for the EBV variations in the direct component of social dominance over time calculated using MCMC replicates of average cohort breeding values. **Figure S2.** Distribution of the regression coefficients (slopes) for the EBV variations in the indirect component of social dominance over time calculated using MCMC replicates of average cohort breeding values. **Figure S3.** Distribution of the regression coefficients (slopes) for the EBV variations in milk yield over time calculated using MCMC replicates of average cohort breeding values. **Figure S4.** Distribution of the regression coefficients (slopes) for the EBV variations in somatic cell score over time calculated using MCMC replicates of average cohort breeding values. **Figure S5.** Distribution of the regression coefficients (slopes) for the EBV variations in fertility over time calculated using MCMC replicates of average cohort breeding values. **Figure S6.** Distribution of the regression coefficients (slopes) for the EBV variations in fore udder attach over time calculated using MCMC replicates of average cohort breeding values. **Figure S7.** Distribution of the regression coefficients (slopes) for the EBV variations in udder overall over time calculated using MCMC replicates of average cohort breeding values. **Figure S8.** Distribution of the regression coefficients (slopes) for the EBV variations in rear udder attach over time calculated using MCMC replicates of average cohort breeding values. **Figure S9.** Distribution of the regression coefficients (slopes) for the EBV variations in udder width over time calculated using MCMC replicates of average cohort breeding values. **Figure S10.** Distribution of the regression coefficients (slopes) for the EBV variations in thinness over time calculated using MCMC replicates of average cohort breeding values. **Figure S11.** Distribution of the regression coefficients (slopes) for the EBV variations in front muscularity over time calculated using MCMC replicates of average cohort breeding values. **Figure S12.** Distribution of the regression coefficients (slopes) for the EBV variations in thorax depth over time calculated using MCMC replicates of average cohort breeding values.

## Data Availability

The datasets used and/or analysed during the current study are available from the corresponding author on reasonable request.
