# Supplementary material for: Genetic correlations of direct and indirect genetic components of social dominance with fitness and morphology traits in cattle
Source: Genet Sel Evol. 2023 Nov 30;55:84. doi: 10.1186/s12711-023-00845-8 (PMC10687847; doi:10.1186/s12711-023-00845-8)
Supplement: Supplementary file 1 — Additional file 1. In-depth description of the single-trait models for milk yield, somatic cell score, fertility and morphological traits [34, 49–53, 103, 104]. [file 12711_2023_845_MOESM1_ESM.pdf]

ADDITIONAL FILE 1 for

Genetic correlations of direct and indirect genetic components of social dominance with fitness and morphology traits in cattle

TRAITS DESCRIPTION

Milk yield and somatic cells

Data about milk yield were routinely collected by the technicians of the Italian Breeders Association (www.aia.it) and consisted in individual test-day (TD) records of milk, i.e., the daily milk production of a cow (kg) collected during functional controls. Measures of milk starts with parity and continue until the moment of 'dry cow', some weeks later. A sample from each individual milk collection was routinely processed to measure the somatic cells count as somatic cells count/ml. Somatic cells are body-derived cells normally present at very low level in milk and an indication of the inflammatory status of the mammary gland; they typically appear after mastitis events. They are counted with automatic cell counters and this measurement is considered an udder health indicator (as in, e.g., [50]) and is expressed as somatic cell score (SCS; [51]), that is SCS=3+(log2(SCC/100 000 cells/ml)), where SCC is the somatic cells count. This adjustment is performed in order to analyse the trait with a normal distribution. Higher values of SCS (and thus of somatic cells) indicate a detriment in udder health.

Both milk yield and SCS were analysed with a repeatability test-day model, similar to what is currently used in routinely genetic evaluation of the breed [53].

The model included 270435 records of 21530 animals (34921 in the pedigree). It included as fixed effects the lactation number (5 levels, from 1st to 5th parity order), the age at calving at the target lactation (54 classes), the month of the year in which the cow had the calving at target lactation (48 classes), gestation length class

(16 classes of 15-d intervals). The age at calving and the month of the year were fitted as fixed regression coefficients fitted by using 4th order Legendre polynomials. The linear notation of the model is:

$$y_{ijklmnopq} = H_i + HTD_j + \text{fixed} + \sum_{n=1}^{4} \beta \times \mathbf{z_n(t)} + Pe_p + a_p + e_{ijklmnopq}$$

where $y_{ijklmnopq}$ is the TD record q (milk or SCS) of the cow p, H_i is the random effect of the herd identity i of the cow p (962 levels), HTD_j is the random effect of the herd-test-day j (51,644 levels, for the combination of the farm where the testing took place, the classifier identity and the day the testing took place), 'fixed' are the fixed effects mentioned above, β is the fixed regression coefficient fitted with 4th order Legendre polynomials [103], $z_n(t)$ is a vector of covariates of size n (with n = 4) describing the shape of lactation curve at DIM t, Pe_p is the random permanent environmental effect of the cow p (22913 levels), a_p is the random additive genetic effect of the cow p, and $e_{ijklmnopq}$ is the random residual term. In a matrix notation, the model could be written as follows:

$$\mathbf{y = X\beta + W_H H + W_{HTD} HTD + W_D Pe_D + Z_D a_D + e}$$

where **y** is the vector for the trait (milk yield or SCS); **β** is the vectors for systematic fixed effects reported above; **H** is the vector for the random effect of the cows' herd, **HTD** is the vector for the random effect of the herd-test-day, $\mathbf{Pe_D}$ is the vector for the direct random permanent environmental component; $\mathbf{a_D}$ is the vector for the direct additive genetic effect (36123 levels) and **e** is the random residual term. Furthermore, **X**, $\mathbf{W_H}$, $\mathbf{W_{HTD}}$, $\mathbf{W_D}$, and $\mathbf{Z_D}$ are incidence matrices of the proper dimensions.

Fertility

The fertility trait used in our study was the parity-conception interval (PC; in months); i.e., the timespan between a parity and the subsequent conception date. It is the most widely used measure of fertility in animal

breeding (e.g., [49]), with longer intervals associated to less fertile cows due to the possible events like abortion, disease, or missed conception causing the loss of a reproductive season. In this study, PC was achieved from the cows' lactations information routinely recorded by the Italian Breeders Association. We used 51273 records of PC for 16190 animals, with 28501 animals in the pedigree.

The genetic model, in matrix notation, was written as:

$$\mathbf{y} = \mathbf{X\beta} + \mathbf{W_H H} + \mathbf{W_D Pe_D} + \mathbf{Z_D a_D} + \mathbf{e}$$

where **y** is the vector for the trait; **β** is the vectors for systematic fixed effects, including the lactation number (LN; 13 levels, from 1st to 13th parity order), the year of recording (20 levels), the month of the year in which the cow had the calving (12 levels), the gestation length (16 classes of 15-d intervals); **H** is the vector for the random effect of the cows' herd (1000 levels), $\mathbf{Pe_D}$ is the vector for the direct random permanent environmental component (18661 cows); $\mathbf{a_D}$ is the vector for the direct additive genetic effect (31011 levels) and **e** is the random residual term. Furthermore, **X**, $\mathbf{W_H}$, $\mathbf{W_D}$, and $\mathbf{Z_D}$ were incidence matrices of the proper dimensions.

Morphology

We used several different morphological traits (MTs), all routinely collected and provided by the National Breeders Association. Traits for 23380 individuals (44729 in pedigree) were recorded during the annual scoring of primiparous cows for linear type traits. The evaluation for linear type traits [104] is carried out once in the life of cows (at about 3 years) by trained classifiers following a 1-5 points scale with extreme points corresponding to biological extreme values for that trait (see also [52]). Among the 26 MTs routinely collected [52], the present study considered the following seven: i) fore udder attach (size of the fore udder); ii) rear udder attach (distance between skinfolds and the line joining ischium and hock); iii) udder width; iv) udder overall (an evaluation of udder size); v) thinness (general evaluation of bone and muscle structure); vi) thorax depth (distance between the top line of the chest and the chest floor) and vii) front muscularity (muscle mass

around neck and shoulders). These seven MTs were retained because of their role as indicators of a “feminine” or “masculine” morphology as in Sartori et al. [34], with thorax depth and front muscularity usually more associated with fighting prowess. High scores for thinness, udder measurements identify a feminine conformation, whereas high values of front muscularity and thorax depth identify a more masculine bone structure [34]. The genetic model for morphological traits was written in matrix notation as:

$$\mathbf{y} = \mathbf{X\beta} + \mathbf{W_{HYC}HYC} + \mathbf{Z_{D}a_{D}} + \mathbf{e}$$

where **y** is the vector for the target type trait; **β** is the vectors for systematic fixed effects, including the age of cows at first parity (10 classes equally sized from 21 to 48 months of age, and more than 48 months as last class), the number of days at the moment of recording occurred since the calving and thus since the beginning of lactation (days in milk, 7 classes, from 10 to 30 days after calving as first class; then, from 31 to 181 days after calving using 30 days’ intervals; last, more than 181 days after calving); the year at recording (21 classes, from 2000 to 2020); **HYC** is the vector for the random effect of the herd-year-classifier, or the combined effect of herd, year and classifier identity (6409 levels) summarizing the place and moment of evaluation and the identity of evaluator); $\mathbf{a_D}$ is the vector for the direct additive genetic effect (40189 levels corresponding to all the individuals retained in pedigree) and **e** is the random residual term. Furthermore, **X**, $\mathbf{W_{HYC}}$, and $\mathbf{Z_D}$ are incidence matrices of the proper dimensions.
