# Supplementary material for: Genetic correlations of direct and indirect genetic components of social dominance with fitness and morphology traits in cattle
Source: Genet Sel Evol. 2023 Nov 30;55:84. doi: 10.1186/s12711-023-00845-8 (PMC10687847; doi:10.1186/s12711-023-00845-8)
Supplement: Supplementary file 2 — Additional file 2: Table S1. Descriptive statistics of the traits, including the extreme phenotypic values, the phenotypic mean and its standard deviation (SD). Table S2. Variance components of the traits expressed as mean ± standard error (SE) of posterior density intervals of Gibbs sampling estimates, obtained by running single-trait analysis. Table S3. Genetic (σa1a2) and permanent environmental covariances (σpe1pe2), obtained by running bivariate analyses for each pair of traits including social dominance vs. the other traits. Table S4. Heritability estimates for the traits of interest obtained with bivariate analyses. Posterior means for heritability estimates with their respective standard error (SE) and the 95% high posterior density confidence interval (CI) of Gibbs sampling estimates obtained for the traits of interest by running bivariate analyses. Table S5. Regression coefficients (slopes) of the trait variations over time calculated using the average estimated breeding values (EBV) for the newborns of target years (genetic trends) and % of replicates with slope > 0. [file 12711_2023_845_MOESM2_ESM.pdf]

ADDITIONAL FILE 2 for

**Genetic correlations of direct and indirect genetic components of social dominance with fitness and morphology traits in cattle**

SUPPLEMENTARY TABLES

**Table S1**. Descriptive statistics of traits, including the extreme phenotypic values, the phenotypic mean and its standard deviation (SD).

| Trait (unit of measure) | extreme | values | mean(±SD) |
|---|---|---|---|
| Social dominance total (win/lose) | 1 | 0 | 0.5003 (±0.5000) |
| Milk yield (kg) | 46 | 0.2000 | 11.2228 (±3.7337) |
| Somatic cells score (SCS) | 11.1108 | -3.6439 | 1.4822 (±1.8887) |
| Fertility (months) | 0.5476 | 0.0493 | 0.2237 (±0.0725) |
| Morphological traits (points) | Min. (1) | Max. (5) | |
| Fore udder attach | Short | Long | 2.6736 (±0.8865) |
| Udder overall | Poor | Excellent | 2.6741 (±0.7902) |
| Rear udder attach | Low | High | 2.6277 (±0.8041) |
| Udder width | Narrow | Broad | 2.6017 (±0.8293) |
| Thinness | Heavy | Fine | 3.1519 (±0.8300) |
| Front muscularity | Scarce | Developed | 3.3275 (±0.8454) |
| Thorax depth | Shallow | Very deep | 3.2853 (±0.7552) |

**Table S2.** Variance components of traits: permanent environmental variance $\sigma^2_{pe}$, (all traits except morphology traits) additive genetic variance $\sigma^2_a$ (all traits), variance associated to herd ID $\sigma^2_{herd}$ (social dominance, milk yield, SCS, fertility), variance associated to herd-test-day $\sigma^2_{htd}$ (milk yield, SCS), variance associated to the combined effect of herd-year-classifier $\sigma^2_{hyc}$ (morphology traits), residual variance $\sigma^2_r$ (all traits). Covariance of permanent environment components $\sigma_{peD,C}$ (social dominance), covariance of additive genetic components $\sigma_{aD,C}$ (social dominance) and covariance of focal and opponent herd effects $\sigma_{herdD,C}$ (social dominance) are situated in the covariance row. Variance components are expressed as mean ± standard error (SE) of posterior density intervals of Gibbs sampling estimates, obtained by running single trait analysis.

| Trait | | $\sigma^2_{pe}$ | $\sigma^2_a$ | $\sigma^2_{herd}$ | $\sigma^2_{htd}$/ $\sigma^2_{hyc}$ | $\sigma^2_r$ |
|---|---|---|---|---|---|---|
| | | Mean ±SE | Mean ±SE | Mean ±SE | Mean ±SE | Mean ±SE |
| Social dominance | direct | 0.1917 (±0.0189) | 0.1276 (±0.0168) | 0.0676 (±0.0082) | | 1.0016 (±0.0101) |
| | covariance | -0.1895 (±0.0141) | -0.1236 (±0.0139) | -0.0684 (±0.0075) | | |
| | indirect | 0.1944 (±0.0176) | 0.1231 (±0.0167) | 0.0714 (±0.0087) | | |
| Milk yield | | 2.8006 (±0.0764) | 2.3941 (±0.1053) | 0.8318 (±0.0112) | 1.6473 (±0.0990) | 2.9950 (±0.0098) |
| Somatic cells score | | 0.6514 (±0.0160) | 0.2072 (±0.0179) | 0.3737 (±0.0055) | 0.2421 (±0.0169) | 1.9693 (±0.0063) |
| Fertility | | 9.837e-05 (±1.709e-05) | 8.019e-05 (±2.068e-05) | 0.0004 (±2.846e-05) | | 0.0033 (±2.550e-05) |
| Fore udder attach | | | 0.1180 (±0.0089) | | 0.1560 (±0.0056) | 0.4945 (±0.0080) |
| Udder overall | | | 0.0931 (±0.0072) | | 0.1359 (±0.0046) | 0.3843 (±0.0063) |
| Rear udder attach | | | 0.0893 (±0.0072) | | 0.1424 (±0.0047) | 0.4005 (±0.0065) |
| Udder width | | | 0.0937 (±0.0078) | | 0.1386 (±0.0049) | 0.4425 (±0.0071) |
| Thinness | | | 0.0511 (±0.0064) | | 0.1309 (±0.0049) | 0.4957 (±0.0067) |
| Front muscularity | | | 0.1282 (±0.0082) | | 0.1579 (±0.0052) | 0.3926 (±0.0068) |
| Thorax depth | | | 0.1168 (±0.0070) | | 0.1190 (±0.0041) | 0.3158 (±0.0057) |

**Table S3.** Genetic ($\sigma_{a1a2}$) and permanent environmental covariances ($\sigma_{pe1pe2}$), obtained by running bivariate analyses for each trait pair including social dominance vs. the other traits. The posterior means of the Gibbs samples have been reported with the respective standard errors (SE) and the 95% high posterior density confidence interval (CI) of Gibbs sampling estimates.

| | $\sigma_{a1a2}$ | | | | $\sigma_{pe1pe2}$ | | | |
|---|---|---|---|---|---|---|---|---|
| Trait | Direct dominance | | Indirect dominance | | Direct dominance | | Indirect dominance | |
| | Mean<br>±(SE) | $CI^-$<br>$CI^+$ | Mean<br>±(SE) | $CI^-$<br>$CI^+$ | Mean<br>±(SE) | $CI^-$<br>$CI^+$ | Mean<br>±(SE) | $CI^-$<br>$CI^+$ |
| Milk yield | -0.0107<br>(±0.0034) | -0.0171<br>-0.004 | 0.0075<br>(±0.0033) | 0.0010<br>0.0140 | 0.0086<br>(±0.0031) | 0.0026<br>0.0148 | -0.0078<br>(±0.0031) | -0.0139<br>-0.0017 |
| Somatic cells score | 0.0563<br>(±0.0114) | 0.0341<br>0.0776 | -0.0291<br>(±0.0136) | -0.0576<br>-0.0040 | -0.0170<br>(±0.0132) | -0.0426<br>0.0090 | -0.0007<br>(±0.0154) | -0.0303<br>0.0316 |
| Fertility | 0.0013<br>(±0.0004) | 0.0006<br>0.0029 | -0.0018<br>(±0.0004) | -0.0025<br>-0.0011 | 0.0027<br>(±0.0004) | 0.0019<br>0.0034 | -0.0020<br>(±0.0004) | -0.0030<br>-0.0012 |
| Morphological traits | | | | | | | | |
| Fore udder attach | -0.0004<br>(±0.009) | -0.0183<br>0.0168 | -0.0022<br>(±0.0089) | -0.0197<br>0.0153 | | | | |
| Udder overall | 0.0007<br>(±0.0084) | -0.0162<br>0.0165 | -0.0076<br>(±0.0083) | -0.0236<br>0.0088 | | | | |
| Rear udder attach | -0.0114<br>(±0.008) | -0.0270<br>0.0046 | 0.0030<br>(±0.0081) | -0.013<br>0.0184 | | | | |
| Udder width | -0.0095<br>(±0.0083) | -0.0258<br>0.0064 | 0.0017<br>(±0.0083) | -0.0146<br>0.0182 | | | | |
| Thinness | -0.0029<br>(±0.0077) | -0.0177<br>0.0122 | -0.0039<br>(±0.0079) | -0.0195<br>0.0115 | | | | |
| Front muscularity | 0.0361<br>(±0.0091) | 0.0186<br>0.0542 | -0.0414<br>(±0.0092) | -0.0594<br>-0.0237 | | | | |
| Thorax depth | 0.0329<br>(±0.0089) | 0.0163<br>0.051 | -0.0447<br>(±0.0087) | -0.0614<br>-0.0274 | | | | |

**Table S4**: Posterior means for heritability estimates ($h^2$) with the respective standard error (SE) and the 95% high posterior density confidence interval (CI) of Gibbs sampling estimates obtained for traits of interest by running bivariate analyses. Each line includes the estimates for one of the traits of the pair, with the other trait in brackets.

| Trait (other trait) | | $h^2$(±SE) | $CI^-$ | $CI^+$ |
|---|---|---|---|---|
| Dominance direct | (Milk yield) | 0.1265 (±0.0159) | 0.0964 | 0.1595 |
| Dominance indirect | (Milk yield) | 0.1301 (±0.0160) | 0.1002 | 0.1628 |
| Dominance direct | (Somatic cells score) | 0.1302 (±0.0160) | 0.1010 | 0.1626 |
| Dominance indirect | (Somatic cells score) | 0.1295 (±0.0150) | 0.1018 | 0.1610 |
| Dominance direct | (Fertility) | 0.1224 (±0.0151) | 0.0936 | 0.1533 |
| Dominance indirect | (Fertility) | 0.1351 (±0.0149) | 0.1068 | 0.1660 |
| Dominance direct | (Fore udder attach) | 0.1214 (±0.0160) | 0.0909 | 0.1529 |
| Dominance indirect | (Fore udder attach) | 0.1313 (±0.0161) | 0.0990 | 0.1632 |
| Dominance direct | (Udder overall) | 0.1205 (±0.0161) | 0.0899 | 0.1523 |
| Dominance indirect | (Udder overall) | 0.1322 (±0.0163) | 0.1000 | 0.1646 |
| Dominance direct | (Rear udder attach) | 0.1216 (±0.0167) | 0.0898 | 0.1549 |
| Dominance indirect | (Rear udder attach) | 0.1313 (±0.0165) | 0.0997 | 0.1643 |
| Dominance direct | (Udder width) | 0.1212 (±0.0159) | 0.0896 | 0.1526 |
| Dominance indirect | (Udder width) | 0.1320 (±0.0161) | 0.1003 | 0.1636 |
| Dominance direct | (Thinness) | 0.1220 (±0.0157) | 0.0922 | 0.1528 |
| Dominance indirect | (Thinness) | 0.1314 (±0.0159) | 0.1005 | 0.1629 |
| Dominance direct | (Front muscularity) | 0.1257 (±0.0164) | 0.0954 | 0.1588 |
| Dominance indirect | (Front muscularity) | 0.1298 (±0.0161) | 0.0993 | 0.1627 |
| Dominance direct | (Thorax depth) | 0.1229 (±0.0168) | 0.0917 | 0.1569 |
| Dominance indirect | (Thorax depth) | 0.1323 (±0.0165) | 0.0996 | 0.1655 |
| Milk yield | (Dominance) | 0.2233 (±0.0091) | 0.2062 | 0.2418 |
| Somatic cells score | (Dominance) | 0.0591 (±0.0050) | 0.0497 | 0.0696 |
| Fertility | (Dominance) | 0.0269 (±0.0041) | 0.0193 | 0.0352 |
| Fore udder attach | (Dominance) | 0.1654 (±0.0136) | 0.1392 | 0.1927 |
| Udder overall | (Dominance) | 0.1539 (±0.0130) | 0.1291 | 0.1795 |
| Rear udder attach | (Dominance) | 0.1380 (±0.0125) | 0.1134 | 0.1629 |
| Udder width | (Dominance) | 0.1365 (±0.0126) | 0.1123 | 0.1616 |

| | | | | |
|---|---|---|---|---|
| Thinness | (Dominance) | 0.0830 (±0.0108) | 0.0623 | 0.1042 |
| Front muscularity | (Dominance) | 0.1982 (±0.0137) | 0.1709 | 0.2254 |
| Thorax depth | (Dominance) | 0.2368 (±0.0142) | 0.2091 | 0.2646 |

**Table S5**. Regression coefficients (Slopes) for the trait variations over years calculated using the average estimated breeding values (EBVs) for the newborn of target years (genetic trends) and % of replicates with slope > null model of evolutionary change by drift alone. A time interval from 2000 to 2015 was considered. In **bold** percentages above 95% or below 5%.

| Trait | Slope | % of replicates with slope > null model of evolutionary change by drift alone |
|---|---|---|
| Dominance direct | 0.0252 | **100** |
| Dominance indirect | -0.0260 | **0** |
| Milk yield | -0.0014 | 41.6 |
| Somatic cells score | 0.0036 | **95.3** |
| Fertility | 0.0001 | 52.6 |
| Fore udder attach | 0.0004 | 62.1 |
| Udder overall | -0.0006 | 33.9 |
| Rear udder attach | -0.0012 | 38.6 |
| Udder width | -0.0001 | 60.3 |
| Thinness | < 0.0001 | 52.5 |
| Front muscularity | 0.0013 | **99.4** |
| Thorax depth | 0.0014 | **99.8** |
