# Supplementary material for: Genetic correlations of direct and indirect genetic components of social dominance with fitness and morphology traits in cattle
Source: Genet Sel Evol. 2023 Nov 30;55:84. doi: 10.1186/s12711-023-00845-8 (PMC10687847; doi:10.1186/s12711-023-00845-8)

ADDITIONAL FILE 3 for

Genetic correlations of direct and indirect genetic components of social dominance with fitness and morphology traits in cattle

Figure S1-S12. Distribution of regression coefficients (Slopes) for EBV variations in the target trait (blue bars) calculated using MCMC replicates of average cohort breeding values, plotted against null model of evolutionary change by drift alone of the target trait (white bars).

Figure S1. Distribution of regression coefficients (Slopes) for EBV variations in the direct component of dominance over time calculated using MCMC replicates of average cohort breeding values.

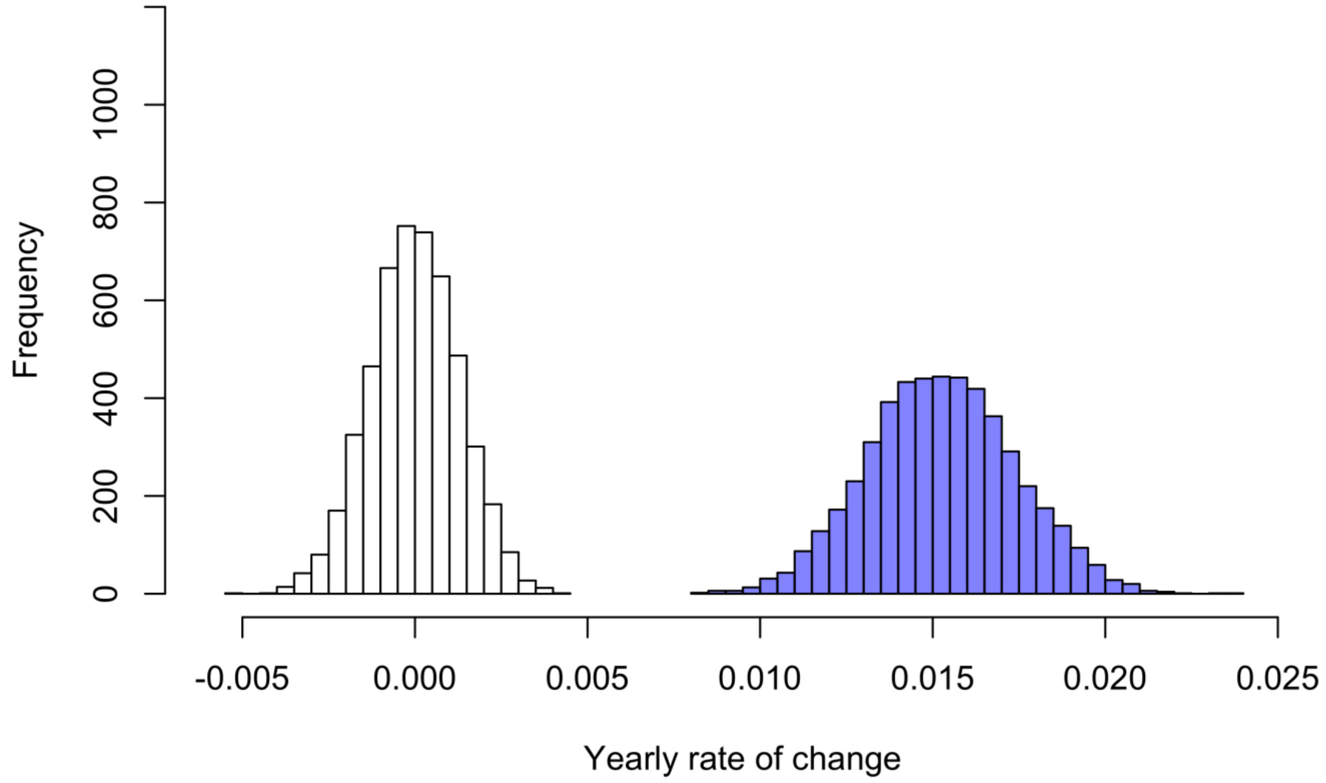

Figure S2. Distribution of regression coefficients (Slopes) for EBV variations in the indirect component of dominance over time calculated using MCMC replicates of average cohort breeding values.

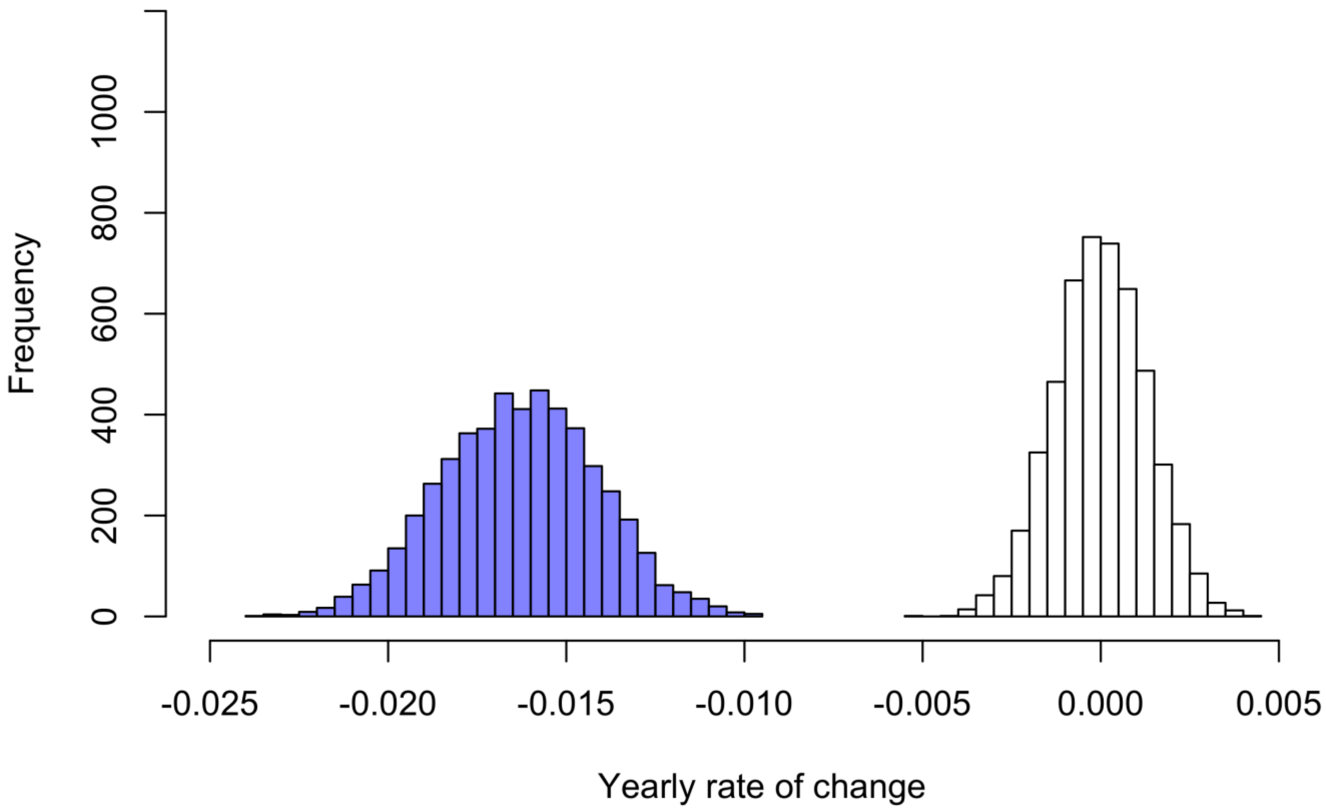

Figure S3. Distribution of regression coefficients (Slopes) for EBV variations in milk yield over time calculated using MCMC replicates of average cohort breeding values.

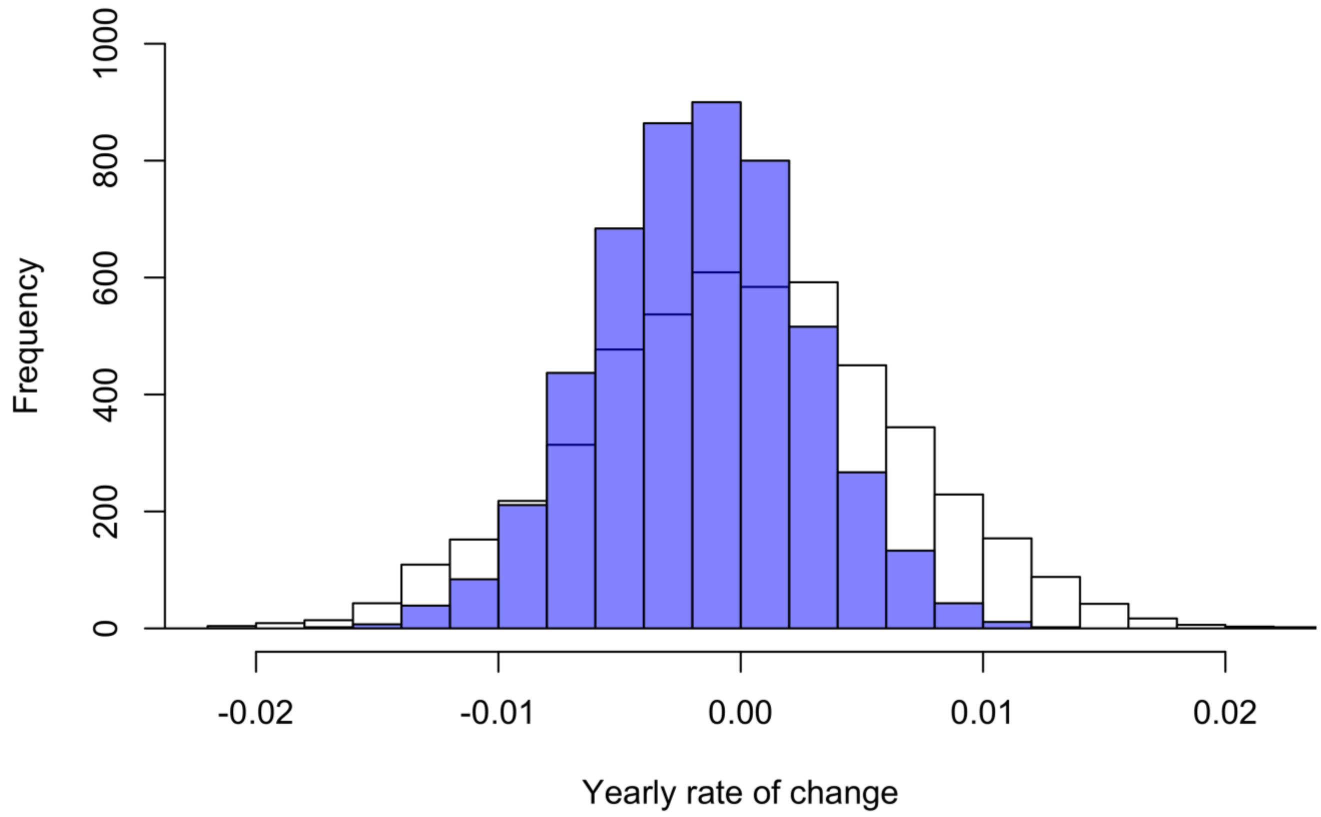

Figure S4. Distribution of regression coefficients (Slopes) for EBV variations in somatic cells score over time calculated using MCMC replicates of average cohort breeding values.

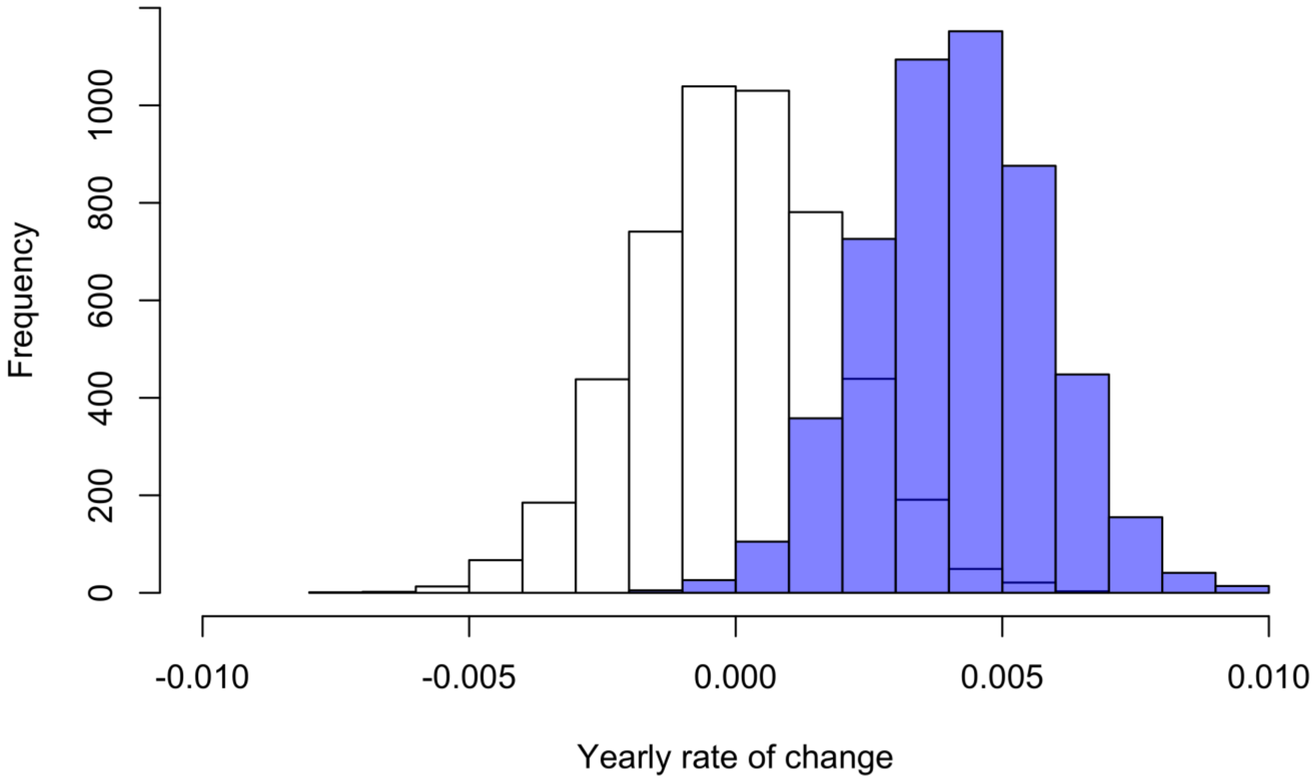

Figure S5. Distribution of regression coefficients (Slopes) for EBV variations in fertility over time calculated using MCMC replicates of average cohort breeding values.

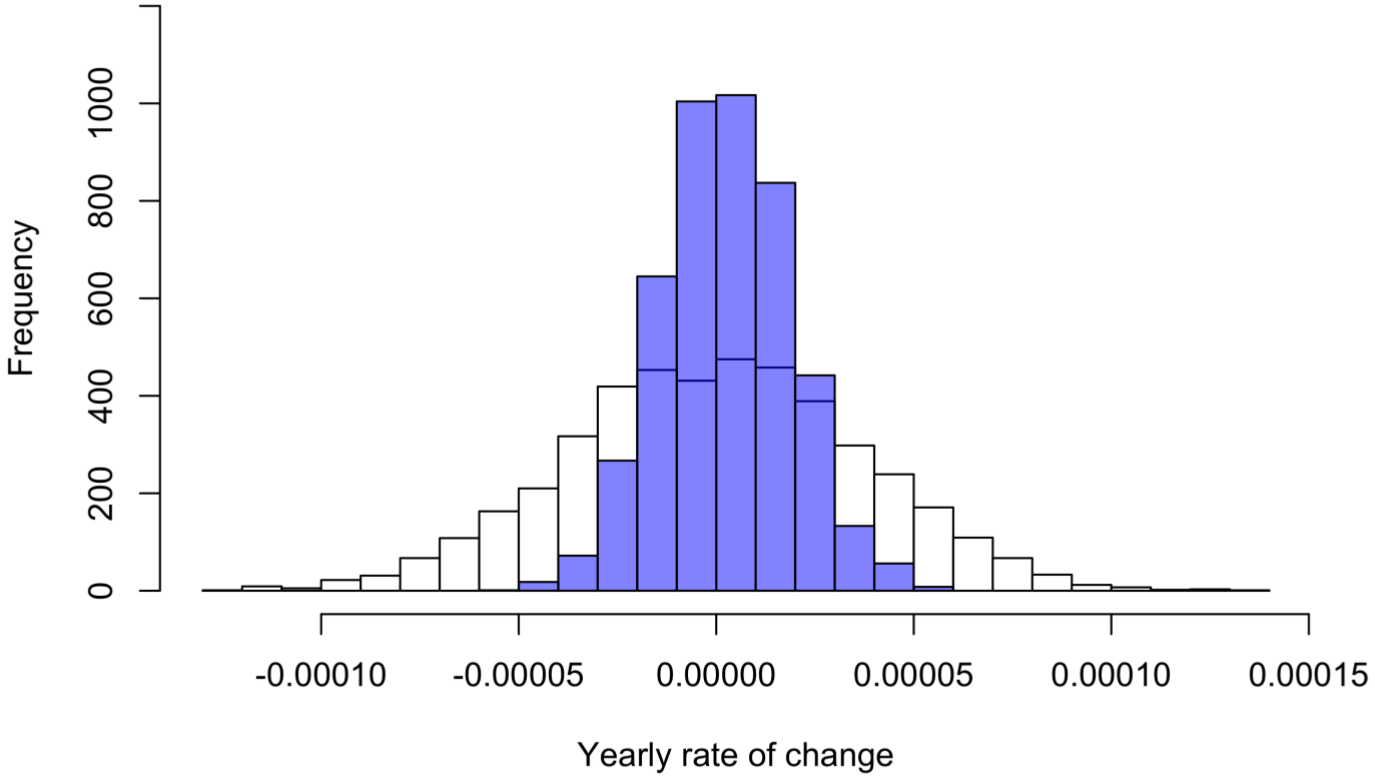

Figure S6. Distribution of regression coefficients (Slopes) for EBV variations in fore udder attach over time calculated using MCMC replicates of average cohort breeding values.

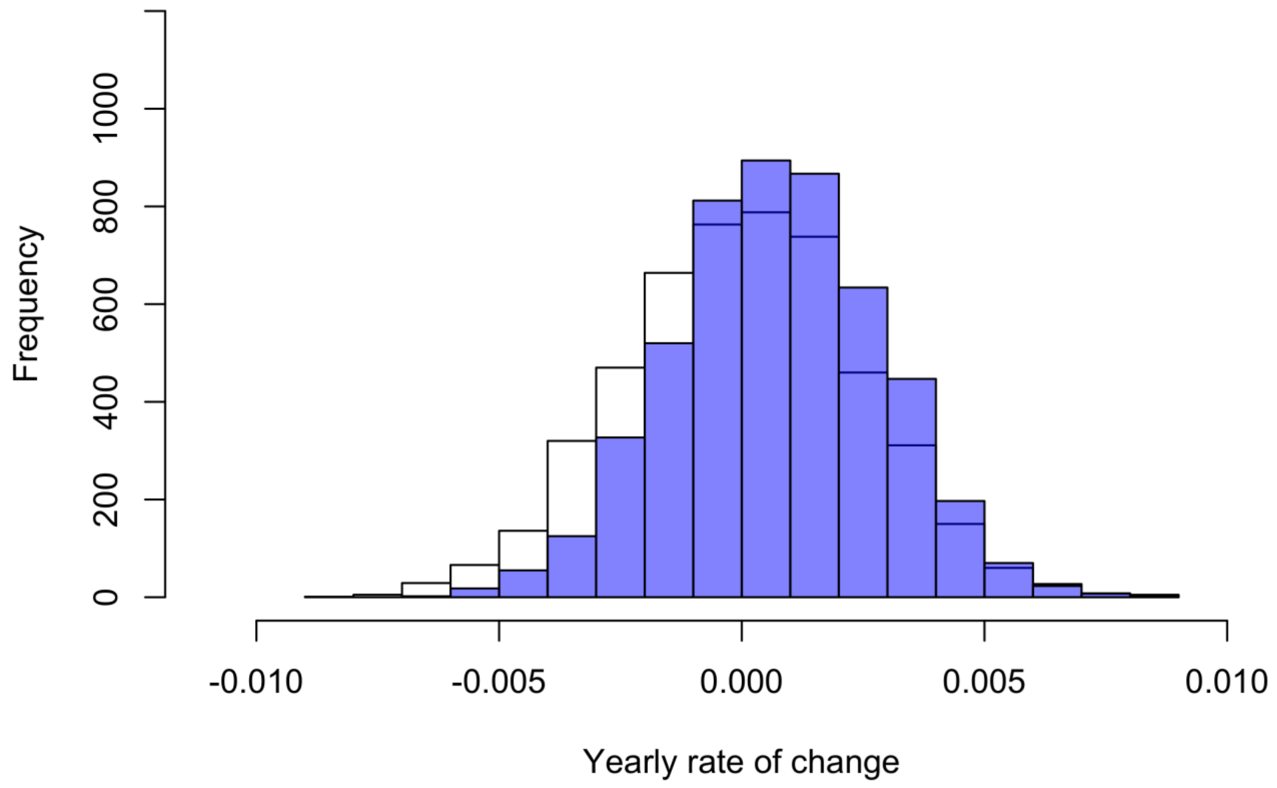

Figure S7. Distribution of regression coefficients (Slopes) for EBV variations in udder overall over time calculated using MCMC replicates of average cohort breeding values.

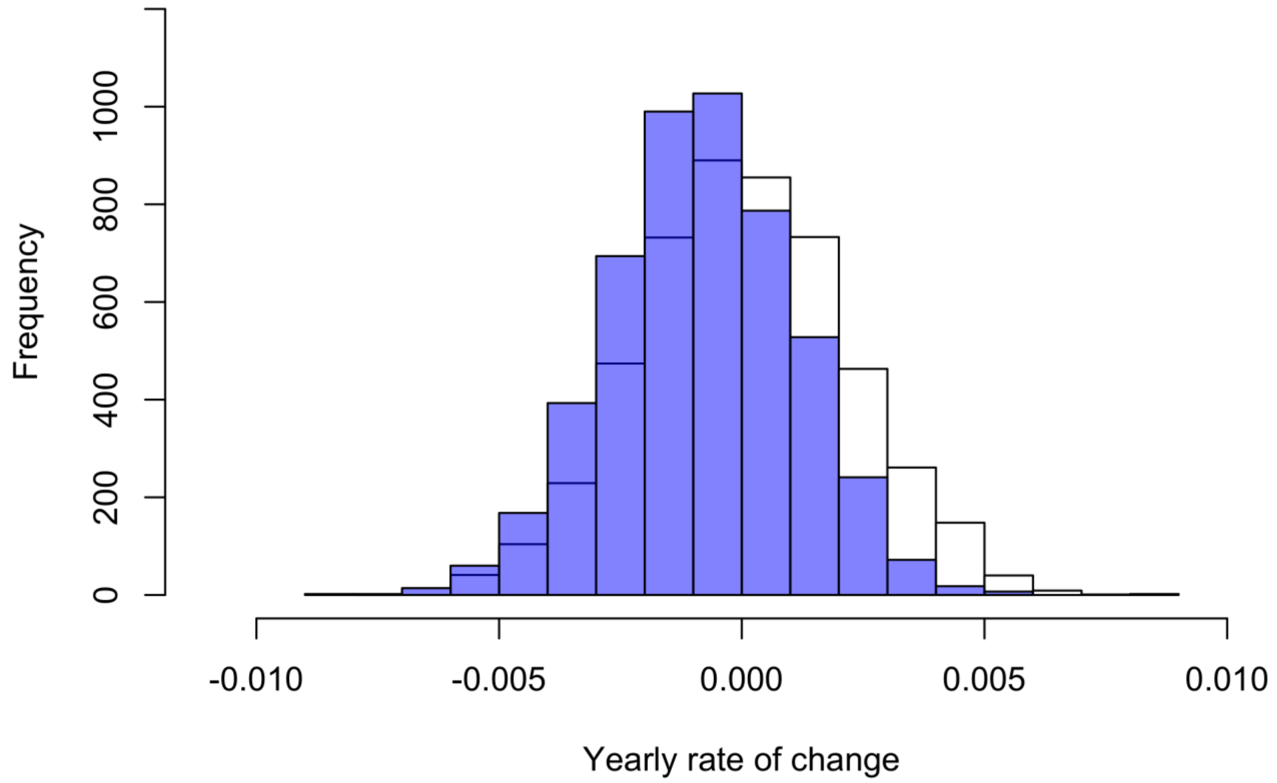

Figure S8. Distribution of regression coefficients (Slopes) for EBV variations in rear udder attach over time calculated using MCMC replicates of average cohort breeding values.

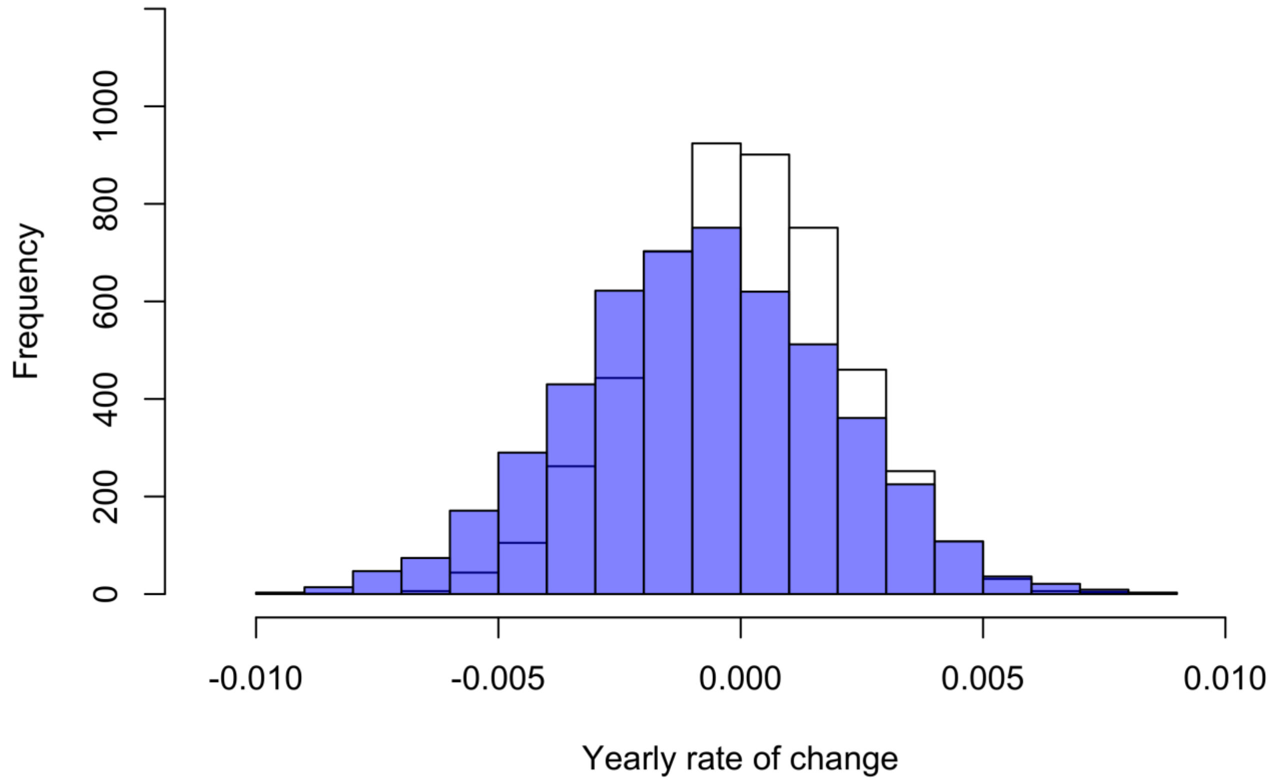

Figure S9. Distribution of regression coefficients (Slopes) for EBV variations in udder width over time calculated using MCMC replicates of average cohort breeding values.

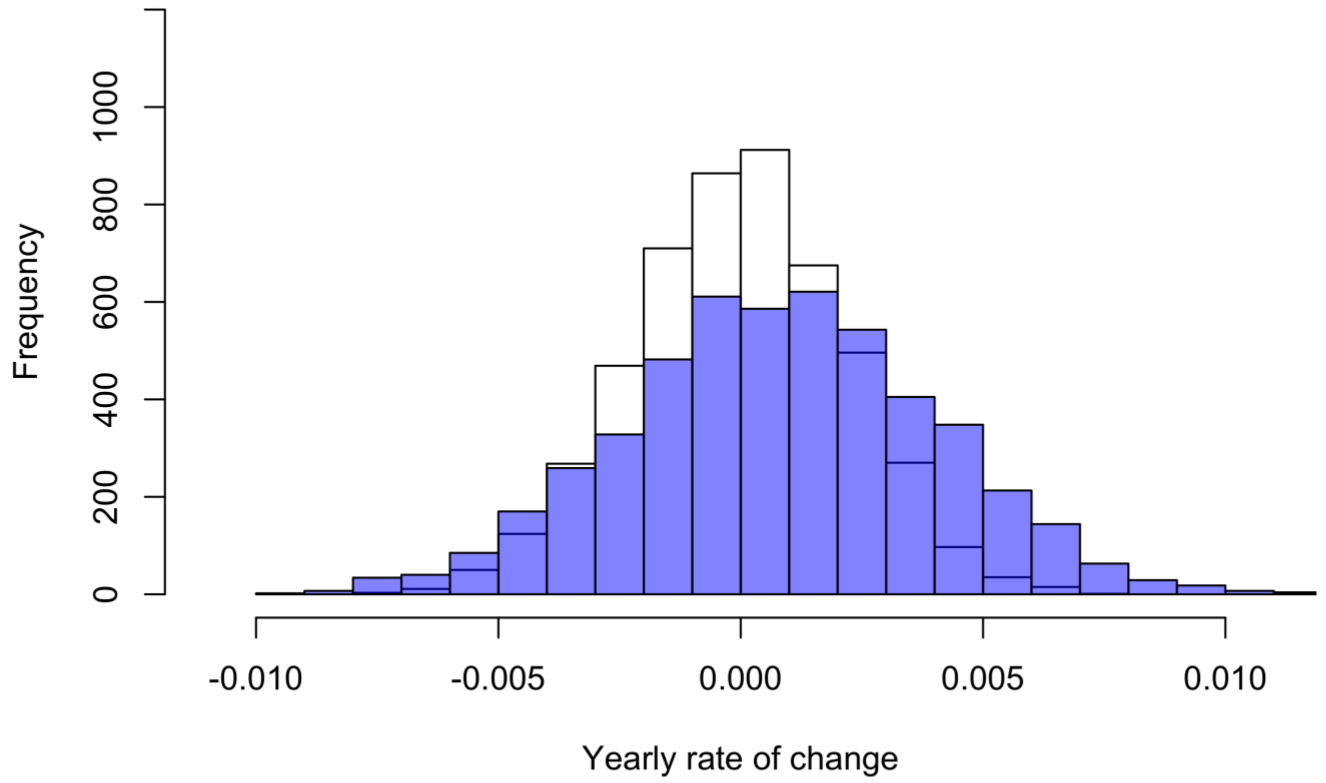

Figure S10. Distribution of regression coefficients (Slopes) for EBV variations in thinness over time calculated using MCMC replicates of average cohort breeding values.

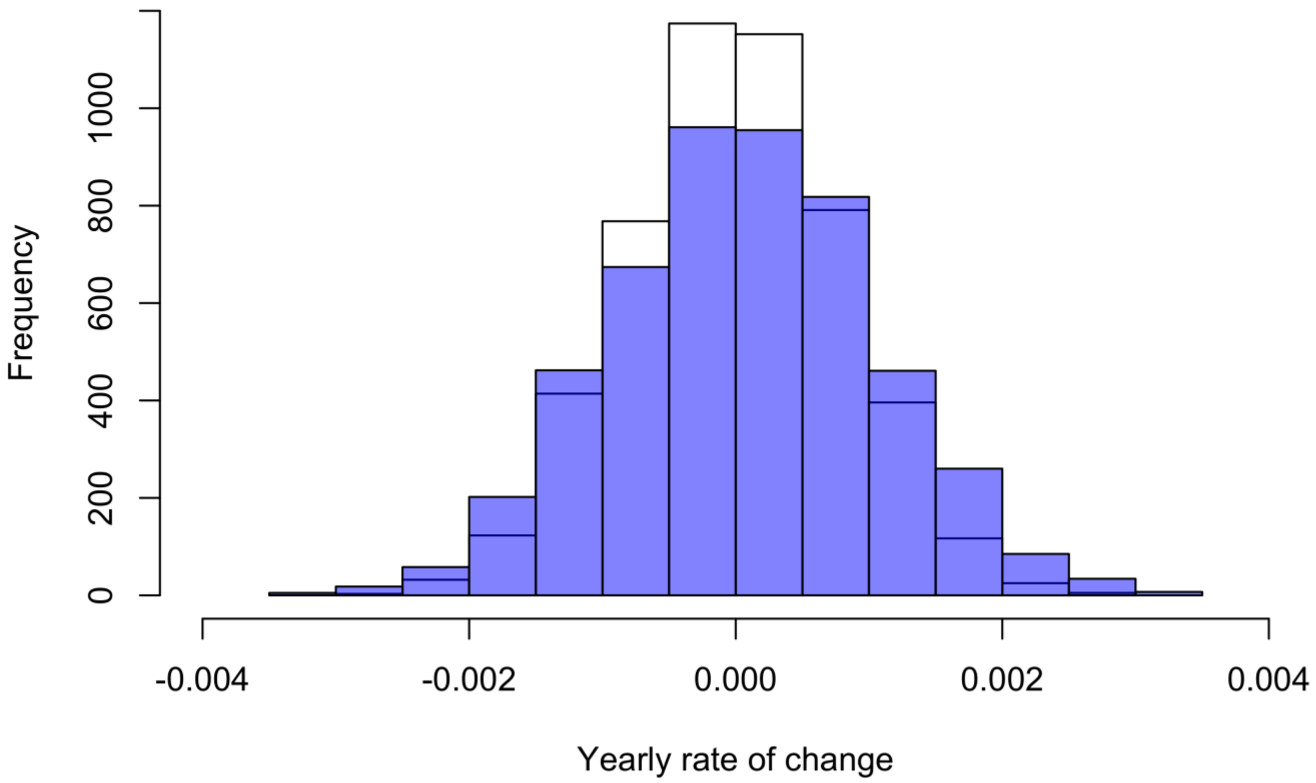

Figure S11. Distribution of regression coefficients (Slopes) for EBV variations in front muscularity over time calculated using MCMC replicates of average cohort breeding values.

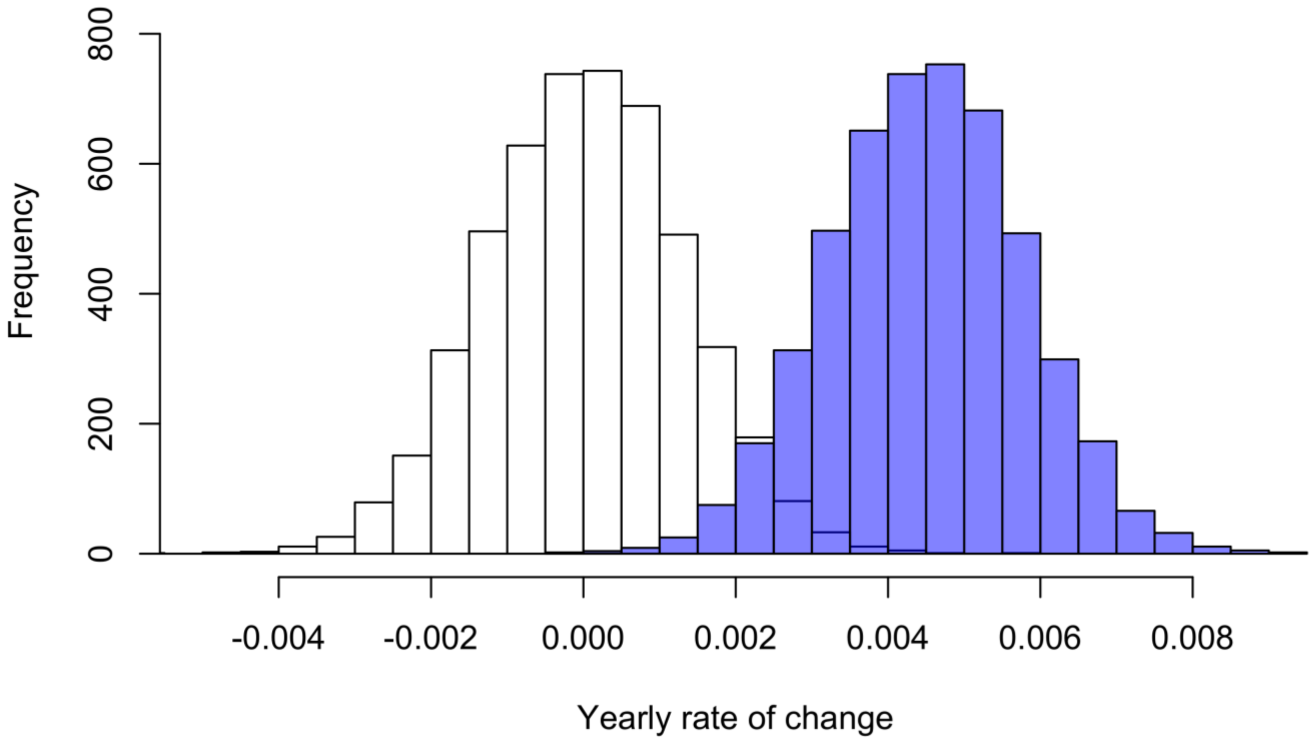

Figure S12. Distribution of regression coefficients (Slopes) for EBV variations in thorax depth over time calculated using MCMC replicates of average cohort breeding values.

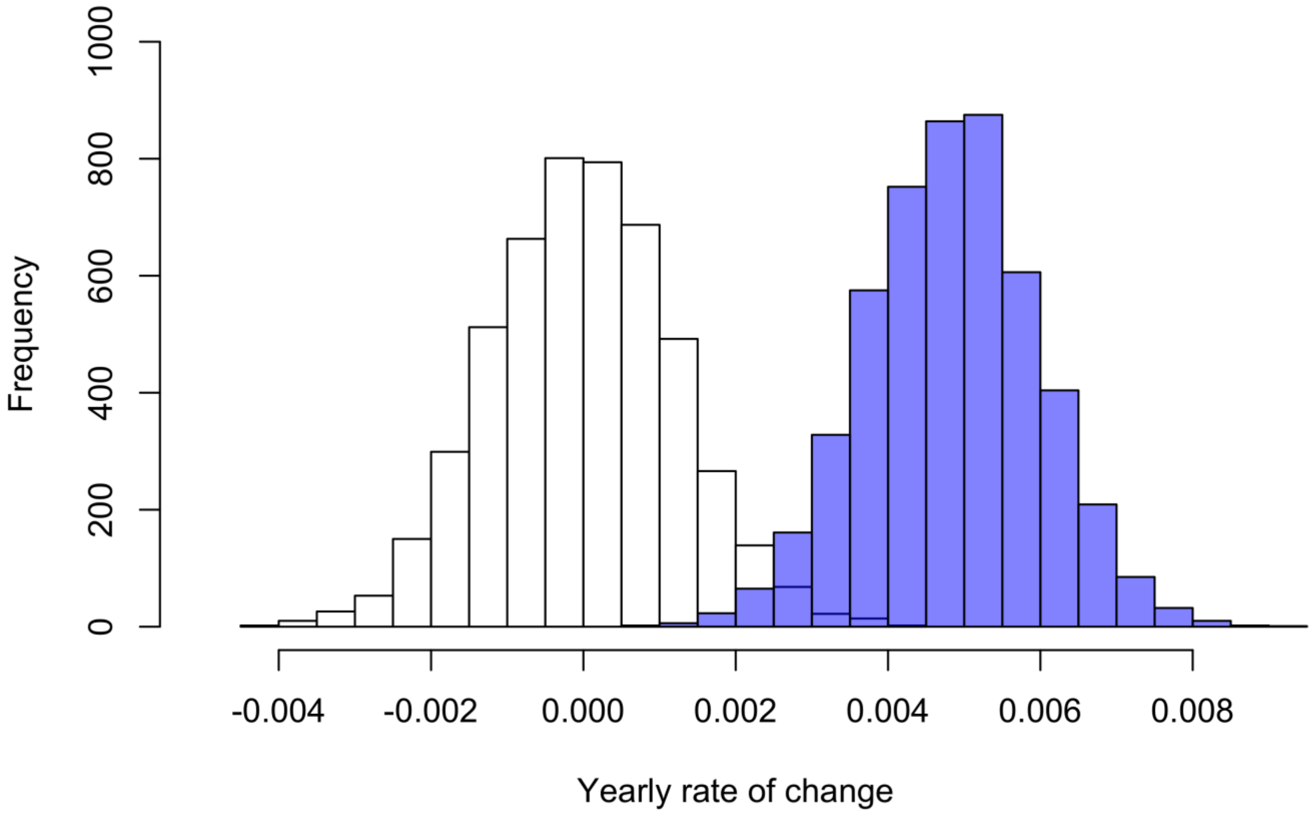

Supplement: Supplementary file 3 — Additional file 3: Figure S1. Distribution of the regression coefficients (slopes) for the EBV variations in the direct component of social dominance over time calculated using MCMC replicates of average cohort breeding values. Figure S2. Distribution of the regression coefficients (slopes) for the EBV variations in the indirect component of social dominance over time calculated using MCMC replicates of average cohort breeding values. Figure S3. Distribution of the regression coefficients (slopes) for the EBV variations in milk yield over time calculated using MCMC replicates of average cohort breeding values. Figure S4. Distribution of the regression coefficients (slopes) for the EBV variations in somatic cell score over time calculated using MCMC replicates of average cohort breeding values. Figure S5. Distribution of the regression coefficients (slopes) for the EBV variations in fertility over time calculated using MCMC replicates of average cohort breeding values. Figure S6. Distribution of the regression coefficients (slopes) for the EBV variations in fore udder attach over time calculated using MCMC replicates of average cohort breeding values. Figure S7. Distribution of the regression coefficients (slopes) for the EBV variations in udder overall over time calculated using MCMC replicates of average cohort breeding values. Figure S8. Distribution of the regression coefficients (slopes) for the EBV variations in rear udder attach over time calculated using MCMC replicates of average cohort breeding values. Figure S9. Distribution of the regression coefficients (slopes) for the EBV variations in udder width over time calculated using MCMC replicates of average cohort breeding values. Figure S10. Distribution of the regression coefficients (slopes) for the EBV variations in thinness over time calculated using MCMC replicates of average cohort breeding values. Figure S11. Distribution of the regression coefficients (slopes) for the EBV variations in front muscular [file 12711_2023_845_MOESM3_ESM.pdf]
